# Senescence of human pancreatic beta cells enhances functional maturation through chromatin reorganization and promotes interferon responsiveness

**DOI:** 10.1093/nar/gkae313

**Published:** 2024-04-29

**Authors:** Milan Patra, Agnes Klochendler, Reba Condiotti, Binyamin Kaffe, Sharona Elgavish, Zeina Drawshy, Dana Avrahami, Masashi Narita, Matan Hofree, Yotam Drier, Eran Meshorer, Yuval Dor, Ittai Ben-Porath

**Affiliations:** Department of Developmental Biology and Cancer Research, Institute for Medical Research Israel-Canada, Faculty of Medicine, The Hebrew University of Jerusalem, Jerusalem, Israel; Department of Developmental Biology and Cancer Research, Institute for Medical Research Israel-Canada, Faculty of Medicine, The Hebrew University of Jerusalem, Jerusalem, Israel; Department of Developmental Biology and Cancer Research, Institute for Medical Research Israel-Canada, Faculty of Medicine, The Hebrew University of Jerusalem, Jerusalem, Israel; Department of Genetics, the Institute of Life Sciences and the Edmond and Lily Safra Center for Brain Sciences (ELSC), The Hebrew University of Jerusalem, Jerusalem, Israel; Info-CORE, Bioinformatics Unit of the I-CORE at the Hebrew University of Jerusalem, Jerusalem, Israel; Department of Developmental Biology and Cancer Research, Institute for Medical Research Israel-Canada, Faculty of Medicine, The Hebrew University of Jerusalem, Jerusalem, Israel; Department of Developmental Biology and Cancer Research, Institute for Medical Research Israel-Canada, Faculty of Medicine, The Hebrew University of Jerusalem, Jerusalem, Israel; Cancer Research UK Cambridge Institute, Li Ka Shing Centre, University of Cambridge, Cambridge, UK; The Lautenberg Center for Immunology and Cancer Research, Institute for Medical Research Israel-Canada, Faculty of Medicine, The Hebrew University of Jerusalem, Jerusalem, Israel; School of Computer Science and Engineering, The Hebrew University of Jerusalem, Jerusalem, Israel; The Lautenberg Center for Immunology and Cancer Research, Institute for Medical Research Israel-Canada, Faculty of Medicine, The Hebrew University of Jerusalem, Jerusalem, Israel; Department of Genetics, the Institute of Life Sciences and the Edmond and Lily Safra Center for Brain Sciences (ELSC), The Hebrew University of Jerusalem, Jerusalem, Israel; Department of Developmental Biology and Cancer Research, Institute for Medical Research Israel-Canada, Faculty of Medicine, The Hebrew University of Jerusalem, Jerusalem, Israel; Department of Developmental Biology and Cancer Research, Institute for Medical Research Israel-Canada, Faculty of Medicine, The Hebrew University of Jerusalem, Jerusalem, Israel

## Abstract

Senescent cells can influence the function of tissues in which they reside, and their propensity for disease. A portion of adult human pancreatic beta cells express the senescence marker p16, yet it is unclear whether they are in a senescent state, and how this affects insulin secretion. We analyzed single-cell transcriptome datasets of adult human beta cells, and found that p16-positive cells express senescence gene signatures, as well as elevated levels of beta-cell maturation genes, consistent with enhanced functionality. Senescent human beta-like cells in culture undergo chromatin reorganization that leads to activation of enhancers regulating functional maturation genes and acquisition of glucose-stimulated insulin secretion capacity. Strikingly, Interferon-stimulated genes are elevated in senescent human beta cells, but genes encoding senescence-associated secretory phenotype (SASP) cytokines are not. Senescent beta cells in culture and in human tissue show elevated levels of cytoplasmic DNA, contributing to their increased interferon responsiveness. Human beta-cell senescence thus involves chromatin-driven upregulation of a functional-maturation program, and increased responsiveness of interferon-stimulated genes, changes that could increase both insulin secretion and immune reactivity.

## Graphical Abstract

**Graphical Abstract:**
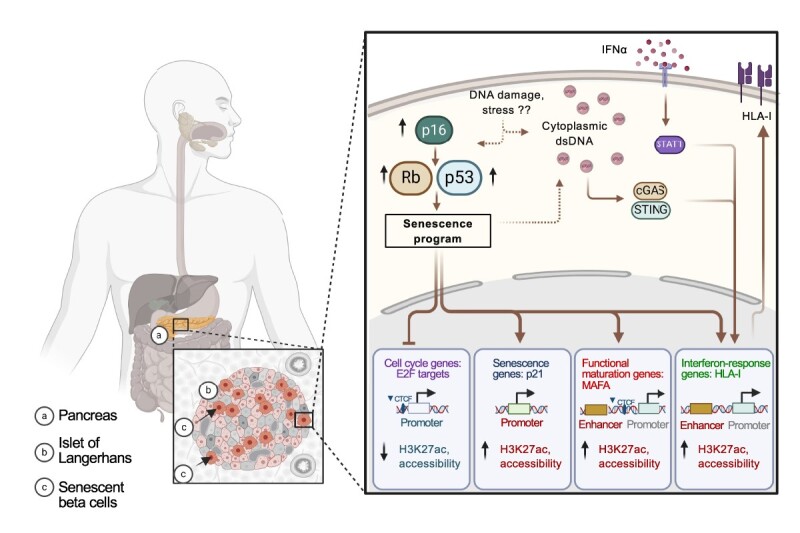

## Introduction

Cellular senescence is a coordinated program activated in cells in situations of damage and oncogenesis, as well as in specific physiologic settings (1–3). The hallmarks of senescence are a permanent cell-cycle arrest, accompanied by a host of morphologic and metabolic changes, and, in some settings, a senescence-associated secretory phenotype (SASP), which involves secretion of inflammatory cytokines. Senescence is typically activated by the Rb and p53 pathways, which induce the silencing of cell-cycle genes (E2F targets), combined with signals that drive the SASP and inflammatory responses (1–3). The senescent phenotype is typically acquired over several days, during which cells undergo chromatin reorganization which drives gene expression reprogramming (4).

Cells displaying features of senescence have been observed in multiple tissues with increasing numbers during aging, and in the context of various diseases (3,5–7). The direct causes of age-associated senescence are mostly unknown, although it is assumed that cellular stress or damage during aging may trigger this response. Detection of senescent cells within tissues is challenging, as there are few markers specific for this state, and their activation is often context- and cell-type-specific (8,9). These include p16^Ink4a^ (also known as CDKN2A, p16 hereafter) which activates Rb through CDK4/6 inhibition, p21 (CDKN1A) – a p53 target and CDK inhibitor, reduced proliferation, senescence-associated β-galactosidase (SA-βgal) activity and expression of SASP and stress-related pathways (8). There is an ongoing effort to develop transcriptomic signatures to allow detection of senescence in single-cell and spatial datasets (8,9).

There is poor understanding as to how senescent cells, which represent a small fraction of tissue cells even upon aging, affect the function of organs and their propensity for disease. Senescent cells have been shown, mostly in mouse models, to contribute to various pathologies, and this has led to an effort to identify drugs that specifically eliminate these cells (senolytics) or block their detrimental activities (1–3). In some settings, however, senescent cells were shown to play roles associated with specific physiologic processes, including promotion of wound healing, fibrosis resolution and tissue regeneration (10–13), execution of phases of embryonic development (14–16) and functional maturation (17,18).

During aging, pancreatic beta cells show increasing levels of p16, in both mice and humans (17,19–22). This has raised the question of whether p16-expressing beta cells enter a senescent state, whether this affects their key function – glucose stimulated insulin secretion (GSIS), and whether these cells influence the onset and progression of diabetes. We previously showed that transgenic p16 activation in mice induces features of senescence in beta cells, and that, rather than reducing functionality, the senescence program enhances the ability of beta cells to secrete insulin at high glucose levels (17). Consistent with this, p16 inactivation was detrimental to beta cell function. These findings suggested that senescence enhances beta-cell maturation and functionality, specifically promoting insulin secretion levels upon exposure to glucose (23). In contrast, potential negative effects of beta cell senescence were described in the context of diabetes. In the NOD mouse model of type I diabetes (T1D), cells expressing senescence markers were shown to accumulate upon disease development, and senolytic treatment reduced immune destruction of beta cells and improved glucose tolerance (24). Other work suggested increased beta cell senescence during type 2 diabetes (T2D), contributing to loss of functionality and disease-promoting inflammation (25). The functional characteristics of senescent beta cells, and the molecular processes that establish them, particularly in humans, therefore require further study.

Here, we set out to elucidate the gene expression programs that characterize senescent beta cells in adult humans, and uncover whether these cells display evidence of altered functionality and enhanced inflammatory features. We dissected the changes in chromatin organization that occur upon senescence of beta-like cells in culture, to identify the key features leading from senescence to changed functionality. Our results establish a strong link between the senescence program in beta cells and activation of functional maturation genes. Furthermore, we highlight a previously uncharacterized aspect of beta cell senescence – increased interferon responsiveness, which is associated with elevated cytoplasmic DNA.

## Materials and methods

### Human samples

Formalin-fixed paraffin-embedded tissue sections of human pancreatic tissues were obtained from the Network for Pancreatic Organ Donors with Diabetes (nPOD). Sections from 11 non-diabetic individuals, aged between 4 months and 75 years were analyzed. Freshly isolated human islets from non-diabetic cadaveric donors were procured from the Integrated Islet Distribution Program (IIDP), as well as from Prodo Laboratories, USA, under their respective ethical consent. Information on all human subjects is provided in Supplementary Table S1.

### Analysis of scRNA-seq data

Single-cell RNA-seq data of pancreatic islets were obtained from Stuart *et al.* (26) (Supplementary Table S2). The datasets were normalized and integrated using the Seurat v3 FindIntegrationAnchors function, followed by the IntegrateData function with 1 to 30 dimensions, after selecting 2000 variable genes for each sample. Expression profiles only of beta cells were filtered for further analysis. Cells having 1000–9000 detected genes, UMI ≥ 500, mitochondrial ratio <0.20, and log_10_ of genes per UMI >0.80 were included. Genes whose expression was greater than 3.5 in at least 2% of beta cells were included. Expression (*Ex*) was calculated as log_2_((CPM/10)+1), where CPM is counts per million reads for each gene. A second independent dataset was obtained from Shrestha et al. (27) and included 10787 beta cells from five non-diabetic donors profiled by 10x Genomics. Beta cells expressing at least 200 genes or more, UMI ≥200, mitochondrial ratio <0.10, and log_10_ of genes per UMI >0.80 were included. We applied the SAVER method (28) to both datasets, to correct for partial transcript capture. Scaled expression values (*E*_s_) were derived from log_2_ SAVER-imputed expression estimates using the ScaleData() function. Gene-to-gene correlations of expression across individual cells were calculated by the SAVER inbuilt function cor.genes(), producing ranked correlations of all genes to p16 (Supplementary Table S3). Genes ranked by their correlation values underwent gene set enrichment analysis (GSEA) against MSigDB global hallmark sets (29) and curated gene sets (Supplementary Table S4). The p16-associated signature includes the 100 genes ranked as highest correlated to p16 expression, and the p21-associated signature includes the top 100 genes correlated to p21. Similarly, the signatures of 250 and 500 highest correlated genes to p16 were tested as p16 Top 250 and 500. All gene signatures and their corresponding sources are listed in Supplementary Table S5. Signature scores were calculated for individual beta cells by the AddModuleScore() function of Seurat. The signature score (SC) is the average scaled expression (*E*_s_) of a gene set (G), subtracting the aggregated expression of the control gene set (C), to correct for confounding cell complexity: SC = average[*E*_s_(G)] – average[*E*_s_(C)]. A control gene set was matched to each gene set to represent comparable expression distribution. All pairwise Pearson correlations between signature scores across all individual cells were calculated using the R cor.test() function, and the generated correlation matrices were plotted using R. We used the Seurat FindMarkers() function with the MAST method (30), fitting two-part, generalized linear models, to identify differentially expressed genes between p16^high^ (>70th percentile) and p16^neg^ (<30th percentile) beta cells. The confounding sources of variation tech (scRNA-seq platforms), and individual (sample donor) were included as covariates (latent.vars). The same method was used to identify differentially expressed genes between cells expressing high (>70th percentile) and low (<30th percentile) scores for the Replicative Senescence signature (Rep Sen^high^ and Rep Sen^low^, respectively). Differentially upregulated genes are shown as log2 fold change, with error bars for individual genes indicating the 95% confidence interval, calculated by 150 cycles of random subsampling of 500 cells from each category with replacement. The differentially expressed genes were tested for gene set enrichments using Metascape (31). The Benjamini–Hochberg procedure was used for multiple-hypothesis correction of differentially expressed genes, as well as for gene set enrichments.

### Cell culture

EndoC-βH3 human pancreatic beta-like cells (32) were maintained in DMEM low glucose (1 g/l) supplemented with 2% fatty acid-free bovine serum albumin fraction V (Roche), 10 mM nicotinamide, 5.5 μg/ml, human transferrin, 6.7 ng/ml sodium selenite, 50 μM β-mercaptoethanol, 100 U/ml penicillin and 100 μg/ml streptomycin. The culture plates were pre-coated with DMEM high glucose (4.5 g/l) supplemented with 2 μg/ml fibronectin (Sigma) and 1% ECM (Sigma) and incubated at 37ºC for 1–2 h. Cells were passaged once a week by trypsin digestion. Senescence was induced by activating the CreER recombinase carried by the cells by treatment with 1 μM 4-hydroxy-tamoxifen (4-OHT, sigma) for 3 weeks. For GSIS measurement, cells were kept for 24 h in 2.8 mM glucose media, then pre-incubated for 1 hour in 0.5 mM glucose containing Kreb's Ringer buffer (KRB), 115 mM NaCl, 5 mM KCl, 1 mM CaCl_2_, 1 mM MgCl_2_, 24 mM NaHCO_3_, 10 mM HEPES pH 7.4, 0.2% BSA. Cells were then incubated with 1.5 or 20 mM glucose containing KRB buffer for 1 h to stimulate glucose-induced insulin secretion. Secreted insulin was measured by ELISA (Crystal Chem). IFNα treatment was done using 2000 u/ml for 24 h, followed by RNA collection. The cells were treated with the STING inhibitor H-151 (1 μM) overnight before the collection of RNA samples.

### Tissue-section and cell stains

Paraffin-embedded tissue sections of human subjects were stained using standard immunofluorescence procedures. Briefly, sections were antigen retrieved with Tris-EDTA (pH-9.0) buffer, permeabilized with 0.1% Triton X 100, and blocked with Cas-Block (Applied Bioscience). Sections were incubated with primary antibodies overnight at 4°C, washed, and incubated with fluorophore-conjugated secondary antibodies (Jackson) for 30–60 min at room temperature. EndoC-βH3 cells seeded in microscopy grade chamber slides were fixed with 4% paraformaldehyde, permeabilized with 0.5% Triton X100 in PBS, blocked with CAS-Block for 10 min and 2.5% BSA, and stained with primary antibodies for 1 hour, followed with fluorophore-conjugated secondary antibodies and DAPI nuclear stain. Cytoplasmic DNA was detected with an anti-dsDNA antibody or with the sensitive nucleic acid-binding dye SYBR gold (Thermo Scientific, #11494) following pre-treatment with RNase I (100 u/ml). The following antibodies were used for cell and section staining: p16 (BioSB, #BSB3480), Insulin (Dako, #IR002), Class I HLA (Abcam, #ab70328), Histone H3 (CST, #14269), dsDNA (EMD Millipore, #MAB1293), H3K27me3 (#IE7 clone CMA323 (33), H3K9me3 (Upstate biotech, #07-523) and Ki67 (Abcam, #ab1667). For senescence-associated β-galactosidase activity (SA-βgal), cells were fixed with 0.2% glutaraldehyde followed by incubation at 37°C with 1 mM MgCl_2_ in PBS pH 6.0, 150 mM NaCl, 5 mM K_4_[Fe(CN)_6_] 3H_2_O, 5 mM K_3_[Fe(CN)_6_], 1 mg/ml X-gal.

### Image acquisition and analysis

Fluorescent images were collected using a Yokogawa CSU-W1 spinning disk field scanning Nikon Ti2 confocal system and a Nikon Ti Eclipse confocal microscope, and processed with NIS-Elements software. Fluorescence signal intensity of p16, HLA-I, and cytoplasmic dsDNA in human pancreatic tissues and EndoC-βH3 cells was quantified using QuPath (v0.4.2) image analysis software. Briefly, all images are loaded to create a QuPath object, followed by annotation of islet regions based on insulin staining fluorescence signal. Each of the cells in the annotated islets was then detected based on DAPI fluorescence, and the ‘cell detection’ tool using cell expansion parameter as 5 μm. In human tissue sections beta cells were classified based on the p16 fluorescence signal as p16^neg^ (<20th percentile), p16^low^ (20–80th percentile) or p16^high^ (>80th percentile). Fluorescence signal for HLA-I and cytosolic DNA were then compared between p16^neg^ and p16^high^ beta cells. For EndoC-βH3 cells, the cells were detected similarly based on DAPI fluorescence, and cytosolic DNA fluorescence was compared between non-senescent and senescent cells. The number of cytosolic DNA foci was scored. Micronuclei were visually identified and scored. The percentages of Ki67^+^ cells and H3K27me3/H3K9me3 heterochromatin foci in EndoC-βH3 cells were scored using ImageJ.

### Flow cytometry of human Islets and cells

Live human islets from non-diabetic human donors were cultured upon arrival in standard Prodo Islet media (PIM(S)) supplemented with 5% human AB blood type serum, glutathione (Prodo), and antibiotic mixture (3×, Prodo) at 37°C overnight. A portion of Islets were treated with IFNα (2000 u/ml) for 24 h before flow-cytometric analysis of cell surface HLA-I. For flow cytometric analysis, the Islets were dissociated gently after incubation at 37°C for 12 min with ACCUMAX (stem cell technologies, USA) in the presence of DNase (100 μg/ml). Live cell suspensions were incubated with C_12_FDG (33 μM) in PIM(S) complete media at 37°C for 1 h to detect SA-βGal activity, followed by incubation on ice with antibodies against HLA-I (Biolegend, #311409), and NTPDase3 to mark beta cells (a gift from Alvin C. Powers). The flow cytometric data were acquired with BD LSR Fortessa, and analyzed with FCS Express software. Control and senescent EndoC-βH3 cells were treated with IFNα (2000 u/ml) for 24 h, followed by staining with C_12_FDG (33 μM) and HLA-I antibody and similar FACS analysis.

### Western blot

Cells were lysed with cell lysis buffer: 20 mM Tris–EDTA (pH 7.5), 150 mM NaCl, 1 mM EDTA, 1 mM EGTA, 1% Triton X 100, 0.1% SDS, 0.5% sodium deoxycholate, 50 mM NaF, 5 mM β glycerol phosphate, 1 mM Na_3_VO_4_, 5% glycerol, 2 mM PMSF, protease inhibitory cocktail (Merck, #11836153001), and phosphatase inhibitory cocktail (Sigma). Western blot analysis was conducted using standard procedures. The anti phospho-STAT1 (CST, #9167), and total STAT1 (Abcam, #ab47425) antibodies were used.

### Gene overexpression and silencing

For p16 overexpression, senescent and non-senescent EndoC-βH3 cells were infected with a lentivirus expressing the human p16 (pLV-hCDKN2A-GFP-Puro, Vector Builder) or a control GFP expressing lentiviral vector (pRRL-GFP) in the presence of polybrene (1 μg/ml) for 24 h. For shRNA-mediated silencing, senescent and non-senescent cells were infected with pLKO-puro lentiviruses carrying an shRNA against cGAS (Sigma, clone: #TRCN0000149984) or a control scrambled shRNA, in presence of polybrene, as above. After 72 h of infection, RNA from the overexpression or shRNA infected cells was collected. CRISPR-mediated excision of the CTCF binding locus was done using a single-step dual guide system (34). We used Cas-Designer (http://www.rgenome.net/cas-designer/) to design guide RNAs for targeting specific regions (Supplementary Table S6). A pair of guide RNAs separated by the sgRNA scaffold and H1 promoter were cloned using the pScaffold-H1 vector (plasmid #118152, Addgene) and then introduced into the lenti-CRISPRv2 blast vector carrying Cas9 (#83480, Addgene). EndoC-βH3 cells were infected with lenti-CRISPRv2 in the presence of polybrene (1 μg/ml) for 24 h and selected with blasticidin (20 μg/ml). The selected cells were used for senescence induction. Genome editing efficiencies were estimated by sequencing and PCR. All lentiviruses were prepared in 293T cells using the packaging vectors pCMVΔR8.2 and pMD2.G.

### RNA extraction, qRT-PCR and transcriptome profiling

Total RNA from EndoC-βH3 cells was extracted in TRI reagent (Sigma) and isolated by an RNA isolation kit (Zymo Research). RNA concentration was measured using EzDrop 1000 micro volume Spectrophotometer (Blue-Ray biotech Corp). cDNA was synthesized using the iScript cDNA synthesis kit (Bio-Rad) as per manufacturer's protocol using 1 μg of total RNA. The qRT-PCR was performed in 10 μl reactions using iTaq Universal SYBR Green Supermix (Bio-Rad) and a BioRAD CFX Real time system in triplicates. PCR cycling conditions are as follows: initial denaturation at 95°C for 20 s, followed by 45 cycles of denaturation at 95°C for 3 s and annealing/extension at 60°C for 30 s, and a final step at 65°C for 5 s, 95°C for 0.50 s. Primers for qRT-PCR are listed in Supplementary Table S6. Relative expression of target genes was determined using ΔCt and normalized to reference gene Ubiquitin C (UBC). For transcriptome profiling, mRNA from senescent and non-senescent cells (*n* = 6 in two independent batches) was extracted and analyzed using CEL-seq2 (35). 3′ cDNA was synthesized and barcoded, followed by RNA synthesis, amplification by *in vitro* transcription, and library generation for paired-end sequencing. Reads were demultiplexed, quality-filtered and trimmed for adapters and poly-A tail using Cutadapt, aligned with the human genome (HSA GRCh38), and counted using Salmon v0.13.1. Differential gene expression was analyzed using DESeq2 with batch correction, and gene-set enrichment calculations were done using Metascape. The E2F and p53 target genes were obtained from the Hallmark dataset in the MsigDB database. The PDX1 and MAF target genes were defined as the nearest genes bound by these proteins in human islets in previous reports (36). The list of IFN-stimulated genes (ISGs) was obtained from Schoggins *et al.* (37).

### ChIP-seq

ChIP-seq was first conducted for several chromatin modifications using multiplexed ChIP-seq (MINT-ChIP) (38). 500000 non-senescent and senescent EndoC-βH3 cells (*n* = 4 replicates) were collected and processed following the v2 protocol. Briefly, cells from each sample are lysed and digested to yield fragments of 1–5 nucleosomes. The ends of the DNA fragments were ligated to adaptors containing sample barcodes (#index 1). Barcoded DNA fragments from each sample were pooled and split for separate ChIP reactions with H3K4me3 (Abcam, ab8580), H3K27me3 (Millipore, 07-449), and H3K9me3 (Abcam, ab8898) antibodies. The purified DNA was linearly amplified and barcoded for each antibody (#index 2) to generate the MINT-ChIP library. The library was sequenced as paired-end in an Illumina NextSeq 500 instrument. Sequenced libraries were de-multiplexed using the bcl2fastq program. For non-multiplexed ChIP-seq, 5 million non-senescent and senescent EndoC-βH3 cells (*n* = 3 replicates for H3K27ac and CTCF pulldowns, *n* = 2 replicates for H3K4me1 and Rb pulldowns) were cross-linked with 1% methanol free formaldehyde followed by quenching with glycine 1.25 M. Cross-linked cells were lysed using SDS lysis buffer (0.5% SDS, 50 mM Tris–Cl ph-8.0, 10mM EDTA) containing protease inhibitor cocktail (Roche #4693116001), sonicated and precipitated with the following antibodies: H3K27ac (Active Motif, #39034), H3K4me1 (Abcam, #8895), CTCF (CST, #3418) and Rb (CST, #9313). Sonication was calibrated to yield fragment sizes between 200 and 700 bp. Chromatin-immune complexes were pulled down with magnetic protein G beads (CST, #9006) and sequentially washed with RIPA buffer, LiCl buffer and TE buffer. Chromatin was eluted from the complex using elution buffer (10 mM Tris–Cl pH 8.0, 0.1% SDS, 1 mM EDTA, 150 mM NaCl and 5 mM DTT), and digested with RNase A (Thermo, #EN0531) and proteinase K (Sigma, 3115836001). Cross-linking between DNA and protein was reversed by incubation at 65°C with NaCl. The ChIP DNA and the corresponding input DNA were size-selected using SPRI magnetic beads. Libraries were prepared using the KAPA Hyper Prep kit (Roche) and sequenced to obtain 5 million paired-end reads per sample using the Illumina NextSeq 500 instrument.

### ChIP-seq data processing

The raw fastq files for H3K27ac, H3K4me1, CTCF and Rb ChIP-seq experiments were trimmed and filtered for low-quality reads (Phred score < 20) using Trim galore! a wrapper of Cutadapt. FastQC (quality score > 30) was used for quality control. We aligned fastq files of ChIP-seq and MINT ChIP-seq experiments to the human genome (hg38) using Bowtie2, and further processed them with samtools. Duplicate reads were removed using Picard MarkDuplicates for H3K27ac, and H3K4me1, and using samtools rmdp for Rb. We used HOMER findPeaks to call peaks of H3K4me3, H3K27ac and H3K4me1. Peaks of H3K27ac and H3K4me1 were identified relative to corresponding input controls. We used SICER to identify broad peaks of the repressive marks H3K27me3 and H3K9me3. We used MACS2 to identify peaks of CTCF and Rb, with the parameters for CTCF: –format BAMPE –SPMR -B -q 0.01; and the parameters for Rb: –format BAMPE –SPMR -t ‘senescence’ -c ‘non-senescence’ -B -p 0.01. CTCF peaks were identified relative to matched input controls, and senescence-specific Rb peaks were identified by comparison to matched non-senescent samples. All CTCF peaks were filtered for the presence of the CTCF binding motif (JASPAR 2018 MA0139.1) identified by FIMO(MEME), *P*_adj_ <0.05. The differential binding of H3K27ac and CTCF (motif-enriched peaks) was analyzed by DESeq2 from raw read counts of peaks. Coverage tracks were generated by deeptools bamCoverage, with size factors for CTCF and H3K27ac derived from DESeq2. The coverage tracking of Rb ChIP-seq was done relative to corresponding input controls using the deeptools bamCompare function. HOMER findMotifsGenome was used for motif enrichments of H3K27ac binding regions in enhancers and promoters (DESeq2 *P*_adj_ < 0.05, fold change 1.5).

### ATAC-seq

We used an ATAC-seq kit (Active Motif, #cat 53150) to prepare libraries from 100 000 non-senescent and senescent EndoC-βH3 cells (*n* = 3 replicates). Cells were lysed in ice-cold ATAC lysis buffer, and the collected nuclei were resuspended in tagmentation master mix containing Tn5 transposon and incubated at 37°C for 30 min. The tagmented DNA was purified and used for library preparation using indexed primers. Raw paired-end sequence reads were generated by Illumina Nextseq 500, obtaining 40–80 million mapped and filtered reads per sample. The fastq files were trimmed and filtered for low quality reads (Phred score < 20) using Trim galore! powered by Cutadapt with the following parameters (-q 20 –length 20 –paired). Overall data quality and trimming were checked using FastQC. The paired-end reads were aligned to the hg38 human reference genome using Bowtie2 with the following parameters: -X 2000 –qc-filter –fr –mm, resulting in a high alignment rate (>97%). The mapped reads were sorted and indexed using samtools. Reads aligned to mitochondrial DNA were filtered out. Duplicate reads were removed using Picard MarkDuplicates. All mapped reads were shifted by +4 bp and –5 bp for ‘+’ strand and ‘–’ strand, respectively using deeptools alignmentSieve –ATACshift, because of the Tn5 dimer binding and adaptor insertion. These final aligned, filtered (mitochondrial reads), de-duplicated, and shifted BAM files were then used in all downstream analyses except for transcription factor footprint analysis. The sorted BAM files (filtered and shifted) were used for MACS2 peak calling with a threshold of FDR-corrected *P* (*q*) < 0.01 using the Benjamini–Hochberg procedure for multiple-hypothesis correction, with the following parameters (–nomodel –call-summits –keep-dup all –format BAMPE -q 0.01 –SPMR -B -g hs). The read counts under union of ATAC-seq peaks were scored using bedtools multicov for differential chromatin accessibility. We identified differential accessible regions using DESeq2 (*P*_adj_ < 0.05, fold change > 1.5). Coverage files (bigwig) were generated using DESeq2 size factors and the deeptools bamCoverage function with the parameters: normalizeUsing = RPKM, effectiveGenomeSize = 2913022398, binSize = 10 and scaleFactor = 1/DESeq2 size factor. Transcription factor footprint analysis of ATAC-seq data was conducted using TOBIAS (39).

### Promoter and enhancer analysis

Transcription start site (TSS) coordinates and the corresponding gene symbols (GRCh38) were downloaded from the UCSC genome browser. The combination of all three active signals: H3K27ac signals, ATAC-seq signals and H3K4me3 signals spanning ±2 kb of TSS were used to determine active promoters. Genomic loci with overlapping H3K4me1 and H3K27ac signals located outside of promoters were defined as enhancers. Active enhancer coordinates of human islets were obtained from published studies (34,40). Enhancers were linked to the nearest genes by ChIPseeker.

## Results

### p16^high^ human beta cells co-express a senescence signature and genes associated with functional maturation

**Figure 1.**
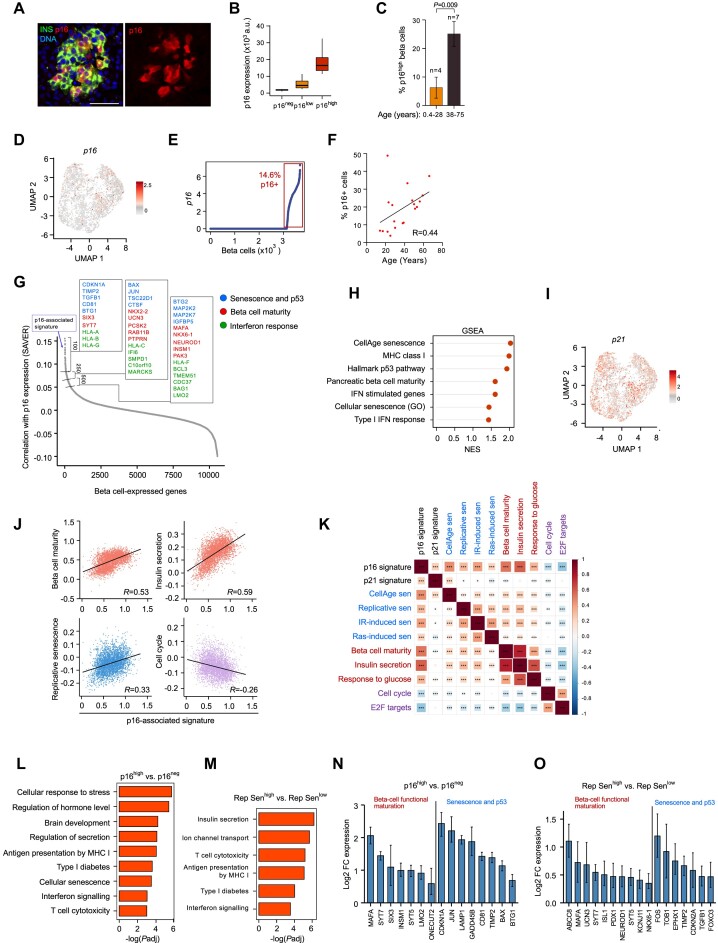
p16^high^ beta cells express genes associated with senescence and functional maturity. (**A**) Pancreatic islets from adult human subject stained for Insulin (INS) marking beta cells, and for p16. Blue (DAPI) marks DNA. Scale bar = 50 μm. (**B**) p16 protein levels in individual beta cells from 5 adult human subjects measured by image analysis of stained sections. Cells are divided into p16^neg^ (bottom 20th percentile) *n* = 804, p16^low^ (20–80th percentile) *n* = 2410, and p16^high^ (top 20th percentile) *n* = 804. y axis fluorescence intensity values represent arbitrary units (a.u.). (**C**) Percentage of p16^high^ beta cells detected in 11 human subjects, divided into age groups as indicated. *t* test. (**D**) UMAP of 3679 single pancreatic beta cells from 31 human subjects included in the analyzed scRNA-seq dataset, showing expression levels of the senescence marker gene *p16* (*CDKN2A*). (**E**) p16 mRNA levels in the same cells, sorted by transcript levels. Values represent log_2_((counts per million/10) + 1). Red rectangle marks cells in which the p16 transcript was detected. (**F**) Percentage of cells with detected p16 transcript expression in patients with known ages in the datasets shown in panel 1D, and in Supplementary Figure S2A. *n* = 19, non-diabetic subjects. *R* indicates Pearson correlation value. (**G**) Plot of all protein-coding genes expressed in beta cells (dots) ranked by the degree of the correlation of their expression levels to p16 across analyzed beta cells, calculated by SAVER. Left brackets indicate the top 100 most correlated genes, defined as the p16-associated signature, as well as the top 250 and top 500 ranked genes. Boxes show representative genes from enriched gene sets, labeled by color. (**H**) Gene-set enrichment analysis (GSEA) of genes ranked by their correlation to p16 expression across cells. x axis indicates normalized enrichment scores (NES) of gene sets in positively correlated genes (*P*_adj_ ≤ 0.1). (**I**) UMAP of the pancreatic beta cells showing expression levels of *p21* (*CDKN1A*). (**J**) Plots indicating the expression score of the p16-associated signature (x axis) in individual beta cells (dots) relative to scores of other indicated gene signatures in the same cells. *R* values indicate Pearson correlation between the signatures across cells. *P*< 10^−55^ for all correlations. (**K**) Matrix of Pearson correlation values between expression scores of indicated gene signatures (as individually plotted in panel J). Matrix colors indicate *R* values as in scale on right. Asterisks indicate *P* values of correlations: *P< 0.05, **P< 0.005, ***P< 0.0005. Gene sets associated with senescence are labeled in blue (IR = ionizing radiation, sen = senescence), gene sets associated with beta cell functional maturation are labelled in red, and gene sets associated with the cell cycle are labelled in purple. (**L**) Upregulated gene sets in p16^high^ relative to p16^neg^ beta cells (top and bottom 30th percentiles, respectively, as defined by SAVER, *n* = 714 each). x axis indicates –log(*P*adj), hypergeometric test (Metascape). (**M**) Upregulated gene sets in cells expressing high levels of the replicative senescence signature (Rep Sen^high^) relative to cell expressing low levels of this signature (Rep Sen^low^, top and bottom 30th percentiles, respectively, *n* = 714). (**N**, **O**) mRNA levels of indicated beta-cell functional maturation genes (left) and senescence and p53-target genes (right) in p16^high^ vs p16^neg^ beta cells (N) and in Rep Sen^high^ versus Rep Sen^low^ beta cells (O). Cells of non-diabetic individuals are included. Values indicate mean of log_2_ fold-change (FC) of senescent versus non-senescent, *P*_adj_ < 0.05 for all comparisons. Error bars indicate 95% confidence interval.

The expression of p16 in beta cells of adult humans has been reported in several studies (17,20–22,41). However, a characterization of the traits associated with p16 expression in beta cells, and a clear association of p16 with senescence of these cells, is lacking. Consistent with these previous studies, staining for p16 of pancreatic sections of adult human subjects, together with Insulin to mark beta cells, revealed the presence of p16-positive beta cells in islets (Figure 1A and Supplementary Table S1). We noted that a range of p16 staining intensities was observed across beta cells; we therefore scored cells expressing the top 20% level of p16 as p16^high^, the bottom 20% as p16^neg^, and the remainder as p16^low^ (Figure 1B). Adults older than 38 years, had, on average, a higher percentage of p16^high^ beta cells (25.1%) than subjects younger than 28 years of age (6.3%), consistent with the association of p16 with age (Figure 1C).

Next, we investigated whether beta cells expressing p16 can be identified in single-cell transcriptomes of adult human pancreata, and set out to characterize the gene expression patterns of such cells. To do this, we analyzed a previously integrated dataset (26) that is comprised of five published scRNA-seq sets from pancreatic islets of 31 adult donors (Supplementary Table S2) (42–46). The dataset includes 14892 individual pancreatic islet cells, of which we identified 3679 beta cells, forming a distinct UMAP cluster of cells expressing the insulin (*INS*) transcript (Supplementary Figure S1A, B).

Among beta cells, expression of the p16 transcript was detected in 14.6% of cells across subjects (Figure 1D,E, Supplementary Figure S1C and Supplementary Table S2). We observed a positive correlation between the percentage of p16-expressing beta cells and age, in a subset of patients whose age was known (Figure 1F and Supplementary Table S2). Three of the original studies comprising the combined dataset included T2D patients (44–46), a total of 8 samples, and the percentage of p16-expressing beta cells was not significantly higher in these samples than in non-diabetic subjects, suggesting that larger patient sample numbers are required for this analysis (Supplementary Figure S1D).

To uncover the gene expression program associated with p16, we calculated the correlation between p16 transcript levels and levels of all other expressed protein-coding genes across individual beta cells. The lower detection rates of the p16 transcript in the scRNA-seq dataset relative to the antibody stain is consistent with previous work indicating that p16 produces a transcript that, in addition to being short, is expressed in low levels yet is very stable (47). Its detection by scRNA-seq methods is, therefore, likely partial. To address this problem and increase the accuracy of the calculation of gene expression correlation to p16, we used the SAVER expression recovery method, which applies imputation to overcome partial gene transcript capture in scRNA-seq datasets (28). Using this method, we ranked all expressed genes based on their correlation of expression with p16 across individual beta cells (Figure 1G and Supplementary Table S3), and conducted gene set enrichment analysis (GSEA) based on this ranking. This analysis revealed that genes positively correlated with p16 are enriched for sets associated with senescence and p53 activation, supporting the notion that cells with high p16 levels are in a state of senescence (Figure 1G, H). Consistent with this, p21, another central senescence marker, was among the top genes whose expression correlated with p16 (Figure 1G and Supplementary Table S3), although it was expressed in a larger proportion of beta cells (64%), suggesting lower specificity (Figure 1I). We found that gene sets associated with beta-cell identity and maturation were enriched among genes correlating with p16, and these included master beta cell regulators such as MAFA, NKX6.1 and SIX3 (Figure 1G,H and Supplementary Table S4). This finding suggests enhanced maturity and function of human p16-expressing beta cells, consistent with our and others’ previous observations (17,48).

In order to establish a robust gene signature that would increase the detection of senescent beta cells, we defined the 100 genes most correlated with p16 as a ‘p16-associated signature’ (Figure 1G and Supplementary Table S5). We calculated the expression score of this gene signature for each beta cell, and, similarly, calculated for each cell the expression scores of additional senescence and beta-cell identity-related gene signatures (Supplementary Table S5). Expression scores represent the average expression of genes in each signature (normalized to matched control signatures). We then tested the degree of correlation between different signatures in their expression across individual beta cells. We found that the expression of the p16-associated signature in individual cells was positively correlated with the expression of beta-cell identity and function-associated signatures, including signatures of beta cell maturity, insulin secretion, and response to glucose (Figure 1J, K). The p16-associated signature was negatively correlated with cell-cycle gene signatures, and was positively correlated with the expression of several senescence signatures, derived previously from fibroblasts undergoing replicative senescence, Ras-induced senescence, or ionizing radiation-induced senescence (49), as well as the CellAge senescence signature (50) (Figure 1J, K). These externally obtained senescence signatures also showed a positive correlation with the beta cell functional gene sets (Figure 1K). These findings indicate that p16 and its associated signature indeed mark beta cells that are senescent, and that beta cell senescence goes hand in hand with increased expression of functional maturation genes.

Signatures comprised of the top 250 or top 500 most correlated genes with p16 gave similar correlations (Supplementary Figure S1E), indicating that these findings are not reflective an arbitrary cutoff choice. Interestingly, a signature comprised of the top 100 genes most correlated to p21 expression, showed poor correlation to signatures of senescence, beta cell functional maturation, or cell cycle (Figure 1K). p21, therefore, although correlated with p16, appears to be expressed more broadly in human beta cells and is less specific as a beta cell senescence marker.

We further validated these results by identifying differentially-expressed genes whose expression was significantly elevated when comparing p16^high^ beta cells to p16^low^ cells, based on the SAVER p16 expression ranking (top and bottom 30^th^ percentile, respectively) (Supplementary Figure S1F, G). We also identified differentially-expressed genes between beta cells with high versus low levels of the replicative senescence gene signature. Both comparisons indicated that the senescent beta cells express elevated levels of genes associated with beta cell differentiation and function, cell senescence and stress (Figure 1L–O). The genes encoding the beta-cell transcriptional regulators *MAFA, SIX3, INSM1*, as well as functional genes such as *KCNJ11* and *UCN3*, were elevated in the senescent beta cells (Figure 1N, O).

We next tested whether these findings are upheld in an independent scRNA-Seq dataset. We analyzed the transcriptomes of 10787 individual beta cells from five subjects, sequenced by 10x Genomics (27). The analysis validated the findings observed in the dataset above. p16 expression was detected in 20.3% of beta cells, and genes whose expression correlated with p16 were enriched for senescence-associated gene sets, as well as beta cell identity gene sets (Supplementary Figure S2). This analysis indicates that our findings in the original compendium hold across datasets and different sequencing methods.

Together these findings indicate that adult humans carry a substantial subset of p16 ^high^ beta cells which display features of senescence, and express elevated levels of genes associated with beta cell functional maturation.

### Senescent beta cells express elevated levels of interferon-response genes, but not a canonical SASP

**Figure 2.**
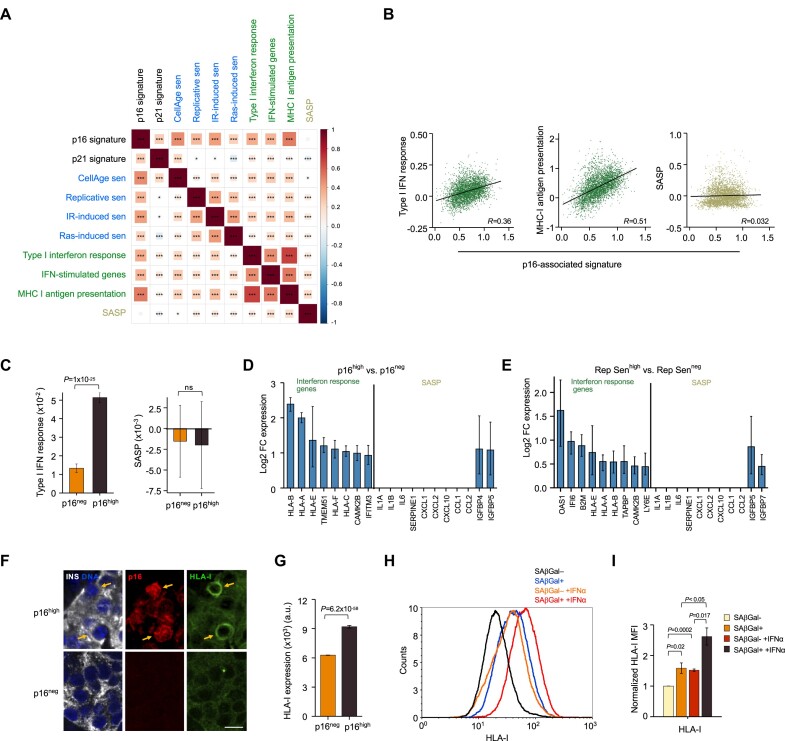
p16^high^ beta cells express elevated levels of interferon-response genes but no SASP. (**A**) Matrix of Pearson correlation values between expression scores of indicated gene signatures in individual beta cells (as analyzed in Figure 1K). Gene sets associated with senescence are labeled in blue, gene sets associated with the interferon response are labelled in green. (**B**) Plots indicating the expression score of the p16-associated signature (x axis) in individual beta cells (dots) relative to scores of other indicated gene signatures in the same cells. *R* values indicate Pearson correlation between the signatures across cells. *P*< 10^−110^ for all correlations, except *P*= 0.05 for the SASP signature. (**C**) Average scores of the IFN-response and SASP signatures in p16^high^ versus p16^neg^ cells. (D, E) mRNA levels of indicated interferon response (left) and SASP genes (right) in p16^high^ versus p16^neg^ beta cells (**D**) and in Rep Sen^high^ versus Rep Sen^low^ beta cells (**E**). Cells of non-diabetic individuals are included. Values indicate mean of log_2_ fold-change (FC) of senescent versus non-senescent. *P*_adj_ < 0.05 for all comparisons. Error bars indicate 95% confidence interval. (**F**) Pancreatic islets from adult human subject stained for Insulin (INS) marking beta cells, for p16, and for HLA-I. Blue (DAPI) marks DNA. Arrows indicate p16^high^ cells. Scale bar = 10 μm. (**G**) HLA-I protein levels in individual beta cells in p16^neg^ and p16^high^ measured by image analysis of stained sections. *n* = 907 per group, from seven subjects. *t* test. (**H**) FACS analysis of dissociated human islet cells obtained live from adult human donor, stained for ENTPD3 marking beta cells, SA-βGal marking senescence, and HLA-I. Plots include only beta cells. Blue and black lines indicate HLA-I levels in the SA-βGal^+^ versus the SA-βGal^–^ cell fraction in the same islets; red and orange lines indicate levels in the same cell fractions in islets treated with IFNα for 24 h. (**I**) Average HLA-I levels in senescent and non-senescent beta cells in human islets obtained from three adult human donors, treated with IFNα for 24 h or untreated, as shown in panel H. Values indicate mean fluorescent intensity (MFI) of samples, normalized to non-senescent untreated cells, ±SEM, *t* test.

Among the genes whose expression correlated positively with p16 in the scRNA-seq datasets were genes associated with the Interferon pathway, most prominently genes involved in MHC class I antigen presentation (HLA-I genes) (Figure 1G,H,L,M). This suggested that senescent beta cells possess elevated IFN pathway activity and antigen presentation, a phenomenon previously described in senescent fibroblasts and tumor cells (51–53) but not in the pancreas. Signatures of Interferon-response genes were positively correlated with the p16-associated signature, as well as with other senescence signatures, and were elevated in p16^high^ cells (Figure 2A–C and Supplementary Figure S2B,D-F). Among the elevated interferon-response genes in the senescent beta cells were genes encoding core components of HLA presentation (HLA-encoding genes, B2M and TAPBP), as well as antiviral effectors IFITM3 and IFI6 (Figure 2D, E and Supplementary Figure S2G).

In contrast, we did not find evidence for SASP activation in the senescent beta cells. A signature comprised of SASP-encoding genes was not correlated with the p16-associated signature or other senescence signatures across individual beta cells, and was not elevated in the senescent beta cells (Figure 2A-C). Expression of genes encoding most SASP cytokines, including *IL1A*, *IL1B*, *IL6* and *CXCL1*, was low to undetectable in most beta cells, and was not elevated in the senescent cell fraction (Figure 2D, E and Supplementary Figure S2F, G). These results indicate that p16^high^ senescent beta cells in human adults do not express a canonical proinflammatory SASP.

We next sought to test whether increased HLA-I protein expression can be observed in senescent beta cells in samples from human subjects. To do this, we co-stained sections of adult human islets with p16 and an antibody against HLA-I proteins (HLA-A,B,C). Image analysis showed that mean HLA-I expression levels were higher in p16^high^ beta cells than in p16^neg^ beta cells (Figure 2F, G). We further verified this finding in live islets isolated from cadavers of human adults (non-diabetic). Dissociated islets from three subjects (Supplementary Table S1) were co-stained for the beta cell marker ENTPD3, the senescence marker SA-βGal, and HLA-I. Strikingly, the SA-βGal^+^ beta cell fraction showed higher levels of HLA-I expression (Figure 2H,I). Furthermore, analysis of islets from the same samples, which were treated with IFNα for 24 h prior to the dissociation and staining, revealed that HLA-I levels were increased in the senescent beta cells to a higher level than in the non-senescent beta cells (Figure 2H, I). Together these results indicate that senescent beta cells do not express a substantial proinflammatory SASP as previously suggested, but display increased levels of HLA-I and other interferon-response genes.

### Senescence of cultured human beta-like cells leads to enhanced GSIS and elevation of functional maturation genes

**Figure 3.**
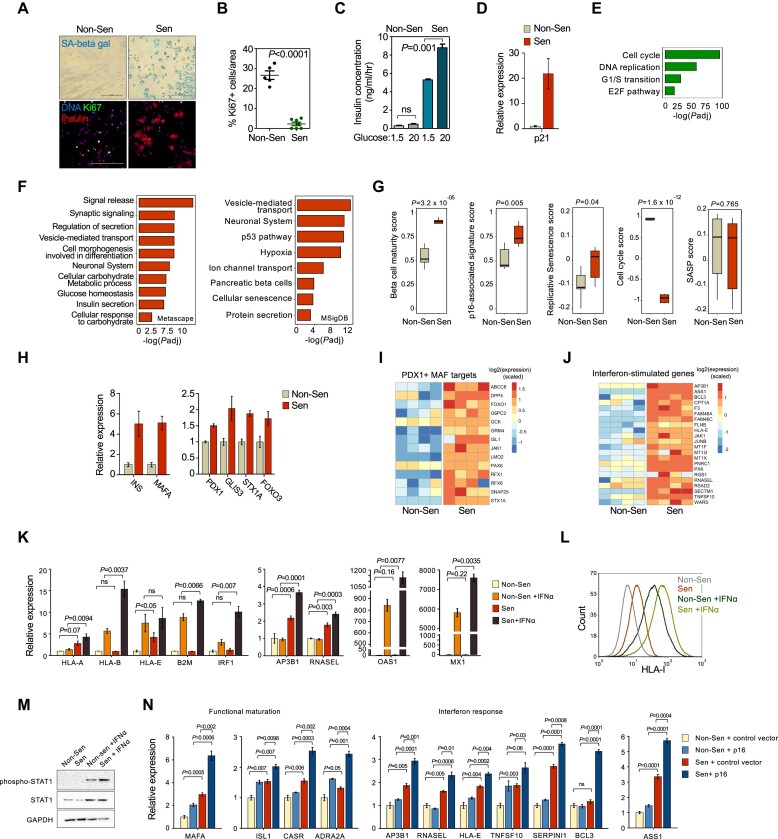
Senescence of cultured human beta-like cells induces functional maturation genes and increases interferon responsiveness. (**A**) EndoC-βH3 cells with or without CreER activation (non-Sen and Sen, respectively) stained for the senescence marker SA-βGal (top, blue) or for Insulin (bottom, red) and the proliferation marker Ki67 (green). Scale bar = 100 μm. (**B**) Percentage of Ki67+ non-senescent and senescent EndoC-βH3 cells stained as in (A). Mean of *n* = 6 replicates ± SEM, *t* test. (**C**) Glucose-stimulated insulin secretion (GSIS) of non-senescent and senescent EndoC-βH3 cells. Insulin levels in medium were measured by ELISA in cells exposed to low (1.5 mM) or high (20 mM) glucose levels. Mean of *n* = 3 replicates ± SEM, *t* test. (**D**) Levels of *p21* mRNA in senescent and non-senescent cells as measured by mRNA-seq. Mean of *n* = 6 replicates ± SEM *P*_adj_ < 0.001, DESeq2. (**E**) Gene sets downregulated in senescent EndoC-βH3 cells. x axis indicates log(*P*_adj_) (Metascape). (**F**) Gene sets upregulated in senescent EndoC-βH3 cells, measured by mRNA-seq, calculated by Metascape (left) or by MSigDB (right). (**G**) Scores of indicated signatures in non-senescent and senescent EndoC-βH3 cells. Boxes indicate interquartile values with indicated median of *n* = 6 replicates, *t* test. (**H**) Relative mRNA levels of indicated genes in non-senescent and senescent EndoC-βH3 cells measured by mRNA-seq. Mean of *n* = 6 replicates ± SEM, *P*_adj_ < 0.001 for all comparisons, DESeq2. (I, J) Heat maps representing scaled log2 expression levels of PDX1 and MAF target genes (**I**) and of interferon-stimulate genes (**J**) in non-senescent and senescent EndoC-βH3 cells (*n* = 4 replicates). (**K**) mRNA levels of indicated interferon-stimulated genes in non-senescent and senescent EndoC-βH3 cells untreated or treated with IFNα, measured by qRT-PCR. Mean of *n* = 3 experimental repeats ± SEM, normalized to untreated non-senescent cells, *t* test. (**L**) FACS analysis HLA-I levels in non-senescent and senescent EndoC-βH3 cells, untreated or treated with IFNα for 24 h. (**M**) Western blot of phospho-STAT1 and total STAT1 levels in non-senescent and senescent EndoC-βH3 cells, untreated or treated with IFNα for 24 h. GAPDH serves as loading control. (**N**) mRNA levels of indicated functional maturation and interferon-stimulated genes in non-senescent and senescent EndoC-βH3 cells expressing a control vector or a p16 overexpression vector, measured by qRT-PCR. Mean of *n* = 3 experimental repeats ± SEM, normalized to control non-senescent cells, *t* test.

The presence of p16^high^ senescent beta cells in human subjects, and their gene expression characteristics uncovered above, supported the hypothesis that senescence modulates beta cell function by reprogramming transcriptional regulation. This called for a detailed dissection of the molecular events occurring upon activation of human beta-cell senescence. The EndoC-βH3 cell line is a unique culture model of human beta cells (32). These cells, derived from a fetal human pancreas, were immortalized through the expression of hTERT and the SV40 Large-T-antigen, which binds and inhibits p53 and Rb (Supplementary Figure S3A). The cells display beta-cell traits and a low proliferation rate. Upon CreER-mediated excision of the immortalizing transgenes, p53 and Rb are released from T-antigen inhibition and, consequently, the cells enter a state of senescence within three weeks (Figure 3A, B), and acquire mature beta-cell function: increased expression of insulin and GSIS capacity (17,32) (Figure 3A, C). This system therefore provides an excellent experimental platform to study causal links between senescence and functional maturation.

To uncover the molecular basis for this link, we first profiled the transcriptome of non-senescent and senescent EndoC-βH3 cells by mRNA-seq. This revealed a large number of differentially-expressed genes: 1647 upregulated and 1745 downregulated (*P*_adj_ < 0.1, fold change > 1.5). The expected activation of p53 upon excision of hTERT and Large T-antigen in the senescent cells was evident through upregulation of p53 target genes, including p21 (Figure 3D and Supplementary Figure S3B), while Rb activation was evident through silencing of E2F targets and cell cycle genes, and increased Rb protein binding to chromatin (Figure 3E and Supplementary Figure S3C, D). p16 protein levels also increased upon Cre activation, with variable levels across individual cells (Supplementary Figure S3E–G). We found that upregulated genes in the senescent EndoC-βH3 cells mirrored those found in the human p16^high^ beta cells *in vivo*, namely gene sets associated with senescence, together with gene sets associated with beta cell functional maturation, including vesicle transport, insulin secretion, glucose homeostasis, carbohydrate metabolism, and neural-like differentiation genes (Figure 3F). The beta-cell maturity signature score was, accordingly, increased in the senescent cells, as were the p16-associated signature derived from the scRNA-seq data, and the replicative senescence signature (Figure 3G and Supplementary Figure S3H). Among the elevated genes were those encoding key regulators of beta-cell identity – *MAFA*, *PDX1* and *GLIS3*, and the insulin-encoding *INS* transcript, which rose ∼4-fold (Figure 3H), as well as previously described direct transcriptional targets of PDX1 and MAF family proteins in human islets (36) (Figure 3I).

A subset of Interferon-response genes was also elevated in the senescent EndoC-βH3 cells, including *HLA-A*, *HLA-E*, *RNASEL*, *AP3B* and others (Figure 3J,K). An elevation in some HLA class II genes was also observed, although their expression was low (Supplementary Figure S3I and data not shown). Treatment of the cells with IFNα revealed that interferon-stimulated genes (ISGs) were induced to a higher level in the senescent cells, and this was observed also in genes that were not elevated in unstimulated cells, such as *HLA-B*, *B2M*, *IRF1*, *OAS1* and *MX1* (Figure 3K). FACS analysis showed that HLA-I protein levels were elevated in the senescent cells, and were induced to a higher level in the senescent cells following IFNα treatment (Figure 3L), similar to our observation in human donor-derived islets (Figure 2H, I). Levels of phosphorylated STAT1, which mediates interferon stimulation, were also elevated in the senescent cells following IFNα treatment relative to non-senescent cells (Figure 3M). These results indicate that beta cell senescence involves an increase in baseline expression of some interferon-response genes, and an increased responsiveness to interferon stimulation, consistent with our observations in the scRNA-seq data and human samples. We did not detect an elevation in the expression of the SASP signature or individual SASP factors in the senescent cells (Figures 3G and S3J).

To test whether p16 itself contributes to the gene expression changes associated with EndoC-βH3 senescence, we infected the cells with a lentivirus overexpressing p16. The p16-overexpressing senescent cells showed higher levels of functional genes, such as *MAFA* and *ISL1*, as well as of IFN-response genes, illustrating that p16 levels drive the senescence-associated phenotypes (Figure 3N). In previous work, we showed that silencing of p16 in senescent EndoC-βH2 cells reduces their GSIS function and other senescence features (17). This indicates that p16 activity promotes beta cell senescence-associated features.

Together, these findings establish that the EndoC-βH3 system is faithful to human senescent beta cells, and demonstrate that activation of functional genes, as well as a heightened interferon response, are inherent to human beta cell senescence, whereas SASP activation is not an obligate feature of this state.

### Senescence causes deactivation of cell-cycle gene promoters and activation of functional gene enhancers

**Figure 4.**
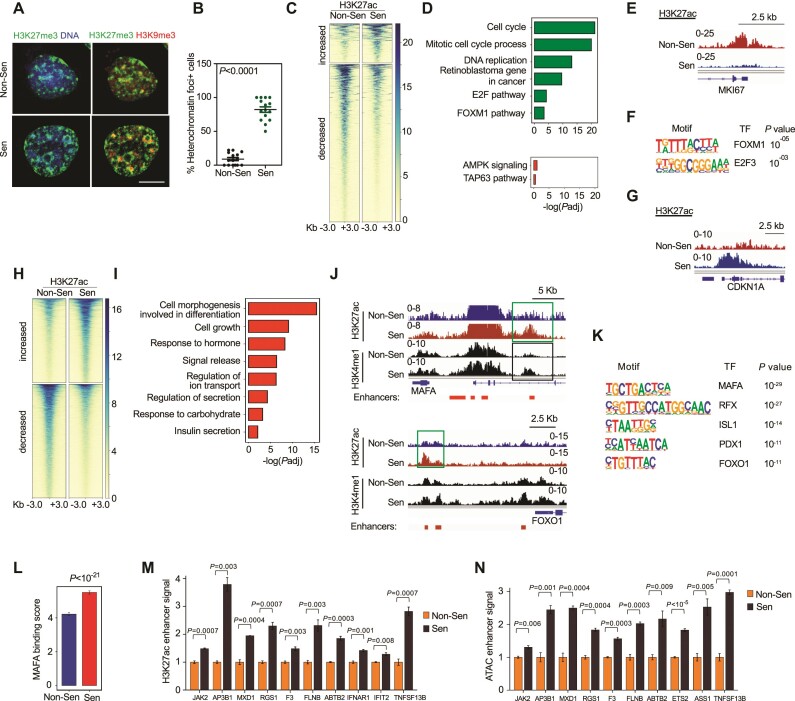
Enhancers of functional maturation and interferon-response genes are activated in senescent beta-like cells. (**A**) Staining of heterochromatin marks H3K27me3 (green) and H3K9me3 (red) in representative non-senescent and senescent EndoC-βH3 cell nuclei. Note heterochromatic foci in the senescent nucleus. Scale bar = 5 μm. (**B**) Percentage of cells containing heterochromatic foci per microscopic field in non-senescent and senescent EndoC-βH3 cells. Mean of *n* = 15 microscopic fields ± SEM, *t* test. (**C**) Binding levels of H3K27ac in gene promoters (rows) showing increased (top, *n* = 232) or decreased (bottom, *n* = 940) levels in senescent EndoC-βH3 cells (fold change > 1.5, *P*_adj_ < 0.05). Shown are promoters in which all three activation markers – H3K27ac, H3K4me3 and ATAC-Seq accessibility – were changed. Panels show 6 kb regions spanning the transcription start sites (TSS). Color scale indicates coverage of H3K27ac binding. (**D**) Gene sets enriched among gene promoters with reduced (green, top) or increased (red, bottom) activation markers in senescent cells (Metascape). (**E**) ChIP-seq traces showing H3K27ac binding on promoter of the *MKI67* cell-cycle gene in non-senescent and senescent cells. y axis shows scaled cumulative read counts. (**F**) Transcription factor binding motifs enriched in promoters with reduced H3K27ac binding in senescent cells. (**G**) ChIP-seq traces showing H3K27ac binding on the promoter of the senescence gene *CDKN1A* (*p21*) in non-senescent and senescent cells. (**H**) Binding levels of H3K27ac in gene enhancers (rows) showing increased (top) or decreased (bottom) levels upon senescence of EndoC-βH3 cells (fold change > 1.5, *P*_adj_ < 0.05). (**I**) Gene sets enriched among gene enhancers with increased H3K27ac binding in senescent cells (Metascape). (**J**) ChIP-seq traces showing H3K27ac binding on enhancers of beta-cell regulator genes *MAFA*, and *FOXO1*. Bottom marks (red) indicate positions of published enhancer loci (Miguel-Escalada *et al.*). (**K**) Transcription factor binding motifs enriched in enhancers with increased H3K27ac binding in senescent cells. (**L**) Transcription factor footprinting analysis using ATAC-seq data, indicating elevated MAFA binding on active enhancers in senescence. (**M, N**) Relative levels of H3K27ac binding (M) and of accessibility (ATAC-Seq, N) in enhancers of indicated interferon-stimulated genes, in control and senescent cells. Values indicated mean of DESeq2 normalized counts relative to non-senescent controls. *n* = 3, ±SEM, *t* test.

Senescence typically involves the reprogramming of chromatin structure to produce a new functional state, a process that takes place over several days (4). Consistent with chromatin rearrangement in senescent beta cells, staining of senescent and control EndoC-βH3 cells revealed a striking spatial reorganization of the distribution of the repressive chromatin marks H3K27me3 and H3K9me3 in the nucleus (Figure 4A, B). As shown previously for senescent fibroblasts (54), these marks were organized in large foci in the senescent EndoC-βH3 cells, as opposed to a more uniform distribution in control cells.

To obtain a molecular view of chromatin state changes in beta cell senescence, we analyzed chromatin accessibility and modifications in control and senescent EndoC-βH3 cells. We found that a large number of gene promoters showed reduced accessibility and activation marks (H3K4me3 and H3K27ac binding) (940 out of 13519 analyzed), whereas fewer (232) showed a change towards an open active state (*P*_adj_ < 0.05, fold change > 1.5) (Figure 4C and Supplementary Figure S4A, B). Enrichment analysis indicated that the deactivated promoters were mostly of cell-cycle-associated genes, often harboring E2F and FOXM1 binding motifs (Figure 4D–F). ChIP-Seq of Rb revealed that, as expected, many of these cell-cycle genes gained Rb binding in the senescent cells (Supplementary Figure S4C). This indicates that upon beta-cell senescence, proliferation genes are widely repressed through reduced chromatin accessibility and activation marks, together with Rb binding of gene promoters. Interestingly, the deactivated promoters mostly did not acquire the repressive marks H3K27me3 and H3K9me3 (Supplementary Figure S4D), suggesting that they may be amenable to reactivation, or that a longer period of senescence is required for establishing a fully silenced state.

Among the genes whose promoters showed increased binding of the activation mark H3K27ac in senescent cells was *p21* (*CDKN1A*) (Figure 4G). However, enrichment analyses of the activated promoters did not identify beta cell functional maturation or interferon-response gene sets (Figure 4D). We therefore studied changes in accessibility and activation of enhancers upon senescence of beta cells. We identified 14886 and 13076 active enhancers in control and senescent EndoC-βH3 cells, respectively, based on overlapping H3K27ac and H3K4me1 ChIP-seq signals (Supplementary Figure S5A). The majority of these (76%) overlapped with active enhancers previously identified in a comprehensive analysis that defined human islet cell enhancers (34,36). We found that 2458 enhancers showed increased H3K27ac binding, indicating activation, in senescent cells, and 3470 enhancers showed reduced H3K27ac (*P*_adj_ < 0.05, fold change > 1.5) (Figure 4H). A strong correlation existed between enhancer H3K27ac binding and chromatin accessibility, as well as the mRNA levels of the nearest genes (Supplementary Figure S5B). Gene sets associated with beta cell function were enriched among genes with increased enhancer H3K27ac binding, and included response to hormone, signal release and secretion, ion transport, and response to carbohydrates sets (Figure 4I). Functional regulators whose enhancers showed increased H3K27ac included *MAFA* and *FOXO1* (Figure 4J), whose mRNA levels were upregulated (Figure 3H). Furthermore, the binding motifs of MAFA, RFX, ISL1, PDX1 and FOXO1 were enriched in activated enhancers (Figure 4K), indicating that the targets of these regulators were subject to enhanced stimulation, as was observed at the mRNA level (Figure 3H, I, N). Transcription factor footprint analysis using ATAC-seq data suggested increased binding of MAFA to enhancers (Figure 4L). As shown above (Figure 3I), the expression of multiple beta-cell regulator and effector genes (*RFX1*, *ISL1*, *LMO2*, *ABCC8*, *ONECUT2*) nearest to MAFA-bound enhancers was elevated. These results indicate that senescence elicits a widespread activation of beta-cell functional maturation gene enhancers. Examination of enhancers of Interferon-response genes revealed increased accessibility and H3K27ac binding in a subset of these enhancers (Figure 4M, N). This suggests that beta-cell senescence entails signals that lead to increased activation of the interferon-response gene enhancers, underlying the increased responsiveness of this pathway to stimulation.

### Reorganization of CTCF binding is associated with altered activity of cell cycle and beta-cell genes

**Figure 5.**
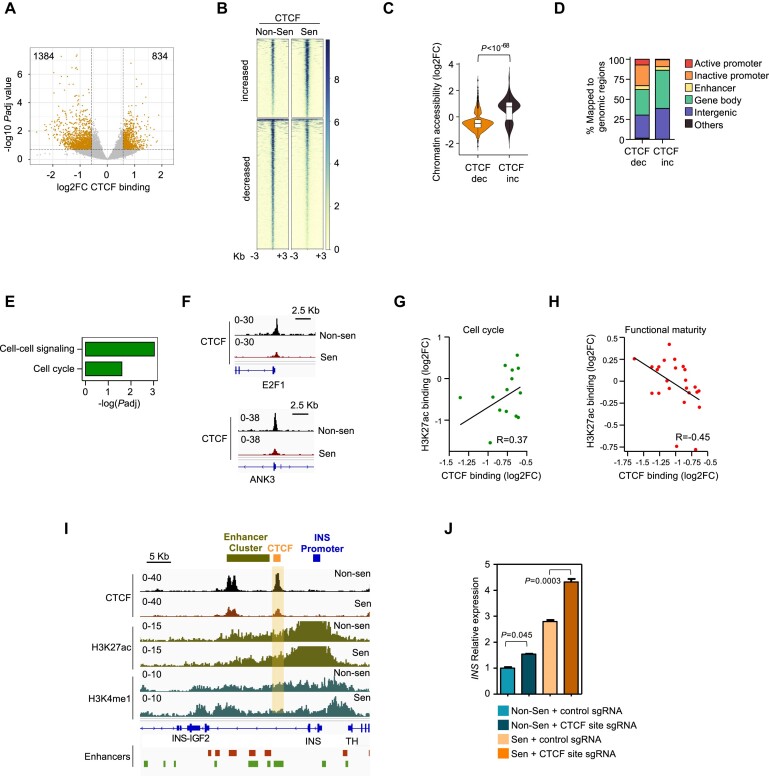
CTCF binding rearrangement in beta-cell senescence. (**A**) Volcano plot of differential CTCF bound genomic loci (dots) in non-senescent and senescent EndoC-βH3 cells (fold change > 1.5, *P*_adj_ < 0.2). Shown are sites that contain a CTCF binding motif. x axis indicates log_2_ fold change of CTCF binding, y axis shows –log_10_*P*_adj_ values. (**B**) CTCF binding levels in genomic sites showing increased (top) or decreased (bottom) binding in senescent cells. (**C**) Chromatin accessibility in regions (±5 kb) of decreased (dec) or increased (inc) CTCF binding. y axis indicates Log2FC of ATAC-seq signal in senescent versus non-senescent cells. (**D**) Percentages of indicated genomic regions among decreased and increased CTCF binding sites in senescent EndoC-βH3 cells. (**E**) Gene set enrichments among genes with reduced promoter CTCF binding in senescent cells. (**F**) CTCF binding traces on the promoters of the *E2F1* and *ANK3* cell-cycle genes. (**G**) Correlation between changed CTCF binding levels (x axis) in sites located on cell-cycle gene promoters (dots) and changed H3K27ac promoter binding. Axes indicate Log_2_ fold change. (**H**) Correlation between CTCF binding change (x axis) in sites (dots) located on TAD boundaries between enhancers and promoters, and H3K27ac binding on promoters of associated beta cell functional-maturation genes. (**I**) CTCF binding traces in the *INS* gene region in non-senescent and senescent cells, relative to H3K27ac and H3K4me1 binding. Red and green marks on bottom indicate previously characterized enhancers (Miguel-Escalada *et al.*, and Greenwald *et al.*, respectively). The CTCF binding site labelled by yellow rectangle is located between the *INS* promoter and a distal enhancer cluster. (**J**) Pre-insulin mRNA levels in non-senescent and senescent cells carrying an sgRNA targeting the region containing the CTCF binding site indicated in panel (I) or a control sgRNA measured by qRT-PCR. Values are mean of *n* = 3 replicates, normalized to control non-senescent cells ± SEM, *t* test.

The CTCF protein plays an important role in the establishment of topologically associating domains (TADs) and in enhancer-promoter interactions (55). To determine whether CTCF binding patterns are changed in senescent beta cells, we mapped CTCF genomic localization in control and senescent EndoC-βH3 cells by ChIP-seq. Examining only sequences that contained the CTCF binding motif, we identified 1384 loci showing reduced CTCF binding in senescent cells and 834 loci gaining CTCF binding (Figure 5A, B). Reduced CTCF binding was associated with reduced chromatin accessibility, and vice versa, suggesting that changes in accessibility may allow or restrict CTCF binding (Figure 5C).

Out of the sites showing changed CTCF binding upon senescence, we found that 24% were located on promoters, and the majority of these showed reduced binding (Figure 5D). Recent studies suggest that promoter-bound CTCF can interact with enhancers and thereby elevate gene expression (56,57). Consistent with this, CTCF binding was preferentially lost from promoters that were active in non-senescent cells (Figure 5D). These promoters were enriched for cell-cell signaling and cell-cycle genes, whose promoter activation state was reduced in the senescent cells (Figure 5E–G). Thus, senescence involves CTCF loss from promoters of proliferation genes upon their silencing, but little gain in binding activated functional-maturation gene promoters.

We examined changes in CTCF binding in sites that were mapped between enhancers and gene promoters, with a maximal range of 200 kb on either side of the CTCF site, and found 822 such CTCF changed sites (37% of total). Of these, 13% (107 sites) were located in boundary regions between previously characterized TADs in pancreatic islets (34). Decreased CTCF binding at these loci could represent disruption of TAD boundary integrity (58). Consistent with this, genes associated with reduced CTCF binding in these sites showed preferential upregulation of gene expression (*P*= 1.36 × 10^−28^, hypergeometric test). Of these, genes associated with beta cell functional maturation showed a negative correlation between CTCF loss and promoter H3K27ac binding (Figure 5H). These findings suggest that CTCF loss in TAD boundary sites may increase enhancer-promoter interactions that contribute to the activation of beta cell functional-maturation genes.

We examined specific CTCF binding sites that could potentially influence beta-cell gene activation. We detected reduced CTCF binding in a site located between the Insulin (*INS*) gene promoter and a previously-described active enhancer cluster (34,40) (Figure 5I). To test whether this CTCF element plays a role in regulating *INS* gene transcription, we conducted dual-guided CRISPR/Cas9 deletion of a 823 bp region that included its binding site in EndoC-βH3 cells (Supplementary Figure S6). We then induced senescence in these cells, and tested the levels of the *INS* pre-mRNA, indicative of gene transcription rates. Cells containing the CTCF site deletion showed elevated levels of *INS* upon senescence (Figure 5J), consistent with the suggestion that this binding site restricts transcription from the *INS* promoter, potentially through insulation from the enhancer cluster.

Together these findings suggest that suppression of cell-cycle genes in senescence is associated with reduced CTCF binding at their promoters, and that lowered CTCF binding at a subset of specific sites located between enhancers and promoters may contribute to activation of beta-cell function genes, including the *INS* gene.

### Senescent beta cells harbor increased levels of cytoplasmic DNA

**Figure 6.**
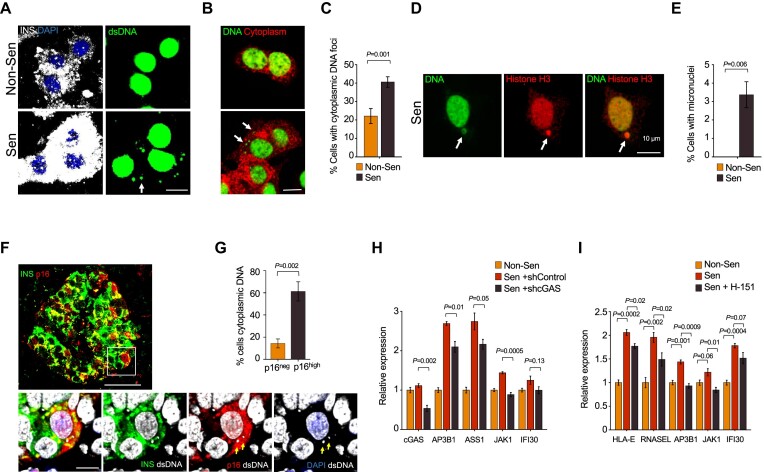
Senescent beta cells harbor elevated levels of cytoplasmic DNA. (**A**) Non-senescent and senescent EndoC-βH3 cells stained with antibodies against Insulin (white, left) and against dsDNA (green, right). DNA foci in the cytoplasms of senescent cells are indicated by arrows. (**B**) Same cells stained with the sensitive DNA dye SYBR Gold indicating cytoplasmic DNA foci (arrows). (**C**) Percentage of non-senescent and senescent EndoC-βH3 cells carrying cytoplasmic DNA foci, as stained in (A). Values indicate mean of 387 non-senescent cells in *n* = 5 microscopic fields and 459 senescent cells in *n* = 17 fields, ±SEM, *t* test. (**D**) Senescent EndoC-βH3 cell with a micronucleus (arrow) stained with the DNA dye SYBR Gold and an antibody against Histone H3 (red). (**E**) Percentage of non-senescent and senescent EndoC-βH3 cells carrying micronuclei, scored as in panel C. (**F**) Section of adult human pancreatic islet stained for Insulin (green), p16 (red), and dsDNA (white). Upper panel shows low magnification of islet (scale bar = 50 μm); lower panels show higher magnification of a p16^high^ beta cell containing cytoplasmic DNA foci (arrows). (**G**) Percentage of p16^neg^ and p16^high^ cells carrying cytoplasmic DNA foci, as stained in (F). Values indicate mean percentage in *n* = 4 subjects ± SEM, *t* test. A total of 506 p16^neg^ and 776 p16^high^ cells were scored. (**H**) mRNA levels of indicated interferon-stimulated genes in non-senescent and senescent EndoC-βH3 cells infected for 3 days with a control shRNA vector, or an shRNA targeting cGAS, measured by qRT-PCR. (**I**) mRNA levels of indicated interferon-stimulated genes in non-senescent and senescent EndoC-βH3 cells, untreated or treated with the STING inhibitor H-151 overnight, measured by qRT-PCR. Graphs in H, I show mean of *n* = 3 experimental repeats ± SEM, normalized to control non-senescent cells, *t* test. Scale bar in A, C, G, E (bottom) =1 0 μm, in E (top) = 50 μm.

We next explored the molecular basis for the elevated interferon responsiveness of the senescent beta cells. Several recent studies in cultured fibroblasts and cancer cells described increased release of DNA to the cytoplasm as a feature of senescence, which can stimulate inflammatory pathways: SASP, Interferon, or both (51,59–61). To test whether this is a feature of beta cell senescence, we stained control and senescent EndoC-βH3 cells with an antibody against dsDNA, or with a highly sensitive DNA dye. Cytoplasmic DNA puncta were clearly detected with both methods, with approximately double the prevalence in the senescent cells (Figure 6A–C). Furthermore, some of the senescent cells, but not control cells, showed the presence of micronuclei—larger DNA foci—which also stained for histone H3, indicating a nuclear source of chromatin (Figure 6D, E). These findings indicate that beta cell senescence entails release of nuclear chromatin fragments to the cytoplasm.

We next tested whether these findings are also evident in human pancreatic tissues. We co-stained sections of pancreata from 5 adult non-diabetic subjects for insulin (to mark beta cells), p16, and dsDNA. Similar to the EndoC-βH3 cells, cytoplasmic DNA puncta were detected in a significantly higher percentage of p16^high^ beta cells than of p16^neg^ cells (Figure 6F, G). This indicates that elevated cytoplasmic DNA release is a feature of senescent human beta cells *in vivo*.

Cytoplasmic DNA is detected by the cGAS-STING complex, which signals to stimulate inflammatory and interferon-response genes (62), and was implicated in mediating the downstream effects of cytoplasmic DNA in senescence (51,59–61). Consistent with this, partial silencing of cGAS mediated by shRNA infection of senescent EndoC-βH3 cells, or treatment of the senescent cells with the STING inhibitor H-151 (63), resulted in reduction in the mRNA levels of several tested interferon-response genes whose levels were elevated in the senescent cells (Figure 6H,I). Overall, these findings indicate that release of DNA into the cytoplasm is a feature of human beta cell senescence in culture and *in vivo*, and may contribute to the sensitization of these cells to the interferon response.

## Discussion

Expression of the senescence activator p16 is detected in pancreatic beta cells in increasing numbers with age (17,19–22). Initial studies in mice have shown that p16 contributes to the non-proliferative state of adult beta cells (19). Our own previous work further demonstrated, using both loss- and gain-of-function mouse models, that p16 induces features of senescence in beta cells, including enhanced glucose uptake, increased mitochondrial activity and increased cell size, and that this results in improved GSIS (17). However, the consequences of p16 activity and senescence in adult human beta-cells, with regards to function, islet inflammation, and diabetes, remain debated.

Here, we characterized p16-expressing beta cells in the human pancreas, and uncovered the molecular mechanisms that underlie senescence-driven gene expression changes. This revealed several fundamental features associated with p16 expression in human beta cells: activation of senescence signatures, elevated expression of functional maturation genes, and increased interferon responsiveness, with a lack of canonical SASP. These features were overall consistently observed in scRNA-seq data, stained human samples, and the EndoC-βH3 culture model. Our data indicate that p16-expressing beta cells are relatively abundant in human adults, often representing 15% of beta cells or more, with their numbers increasing with age. We could not establish whether their numbers are elevated in T2D patients, due to small subject numbers.

Analysis of genes whose expression is associated with that of p16 revealed elevated expression levels of signatures representing beta-cell functional maturation. These included gene sets related to differentiation, protein and ion secretory and transport functions, response to glucose, age-related maturation, and others, reflecting a variety of beta cell features. Concomitantly, the p16^high^ cells showed clear evidence of senescence, reflected in the expression of p53-response genes, p21 and other senescence markers. These striking findings link p16 and senescence to functional maturation in the adult pancreas, rather than to dysfunction.

Analysis of gene expression in chromatin structure in senescent EndoC-βH3 cells revealed that while proliferation-related genes were downregulated by promoter deactivation, beta-cell maturation genes were upregulated largely by gene enhancer activation, driving the expression of master regulators such as MAFA, further activating their targets. Reorganization of enhancers of functional-maturation genes is therefore the central means by which senescence drives beta-cell maturation. Repositioning of CTCF may contribute to both changes in promoter activity and enhancer-promoter interaction, as shown in the case of the Insulin gene.

The increased expression of p16 in mature beta cells could thus represent a physiologic, designated, role for senescence in organismal cell maturation. Senescence was similarly linked to functional maturation of fibroblasts in the context of wound healing or fibrosis (10,11), of liver sinusoid endothelial cells (18), and of macrophages (47). Alternatively, activation of p16 and senescence in beta cells could represent a response to age-accumulated damage or stress. The activation of p53-pathway genes is suggestive of this, and our own previous work has documented DNA damage in beta cells in the context of type 1 and type 2 diabetes (64). Senescence may act in damaged settings to provide enhanced GSIS to compensate for the overall stress.

Mostly absent from the association with senescent beta cells were features of an inflammatory SASP. It has been suggested that senescent cells contribute to both type 1 and type 2 diabetes through the SASP (24,25). Our data clearly indicate that p16-expressing human beta cells do not activate a SASP in the course of aging, and expression of the central known SASP cytokines was, in fact, not detected in the vast majority of beta cells. Activation of a beta-cell SASP may, however, be restricted to specific settings not studied here.

In contrast, we observed elevated levels of HLA-encoding and additional interferon-response genes in senescent beta cells. We detected elevated HLA-I levels in senescent beta cells from human subjects, using section stains as well as stains of cadaver-derived human islets. In the cultured EndoC-βH3 cells, as well as in human islets, increased expression of these genes was observed in response to IFNα treatment. Senescent beta cells may therefore be hyper-responsive to interferon, which could impact their immunogenicity. An enhanced interferon response has been described in several settings of senescence, and interferon signals themselves can also induce senescence (51,65). Therapy-induced senescence of tumor cells was shown to increase their antigen presentation and targeting by the immune system, and this may similarly apply to senescent beta cells (52,53).

Cytoplasmic DNA release has been described in several studies as a source for elevated inflammatory and interferon pathway activity in senescent cells, acting through the DNA sensing complex of cGAS-STING (51,59–61). We found evidence for increased cytoplasmic DNA in senescent human beta cells, in both the culture cell model as well as in the islet tissue sections. This represents a novel association of cytoplasmic DNA with beta cell senescence in particular, and with senescence in adult human tissues in general. Our data support a nuclear origin of this cytoplasmic DNA, since micronuclei showed co-staining for histone H3, yet we cannot fully rule out the contribution of other previously reported sources, such as activation of endogenous retroviral elements or mitochondrial DNA release. Our findings suggest that increased cytoplasmic DNA in senescent beta cells contributes to interferon-pathway gene activation and elevated responsiveness, through cGAS-STING. We did not find evidence of increased interferon secretion by the senescent cells themselves, yet this requires further study. Our findings in the EndoC-βH3 model suggest that cytoplasmic DNA release may be a consequence of senescence rather than a trigger of it, consistent with previous work (51,59,61), although it may also be a consequence of DNA damage that initiates the senescence response.

The potential implications of increased interferon-pathway activation in senescent beta cells, in particular in the pathogenesis of T1D, are of great interest for further research. Elevated interferon responsiveness is a hallmark of beta cells in early T1D (66–68). Beta cell senescence could contribute to this phenomenon, and enhance sensitivity to immune attack. Thus, senescence of beta cells may provide an endogenous, rather than viral, stimulus of the anti-viral response that characterizes islets in early stage T1D.

## Supplementary Material

gkae313_Supplemental_Files

## Data Availability

All datasets were deposited in GEO, under the superseries GSE207826.
