# Correction to ‘Senescence of human pancreatic beta cells enhances functional maturation through chromatin reorganization and promotes interferon responsiveness’

**DOI:** 10.1093/nar/gkae466

**Published:** 2024-05-27

This is a correction to: Milan Patra, Agnes Klochendler, Reba Condiotti, Binyamin Kaffe, Sharona Elgavish, Zeina Drawshy, Dana Avrahami, Masashi Narita, Matan Hofree, Yotam Drier, Eran Meshorer, Yuval Dor, Ittai Ben-Porath, Senescence of human pancreatic beta cells enhances functional maturation through chromatin reorganization and promotes interferon responsiveness, *Nucleic Acids Research*, 2024; gkae313, https://doi.org/10.1093/nar/gkae313

In the originally published version of this article, the following grant numbers were missing from the Funding section: HIRN U01 DK135001 and US NIH grant R01 DK133442 (to Y. Dor) and HIRN U01 DK134995 (to D.A.). These have now been inserted in the HTML and PDF versions of the article.

