# Supplementary material for: Senescence of human pancreatic beta cells enhances functional maturation through chromatin reorganization and promotes interferon responsiveness
Source: Nucleic Acids Res. 2024 Apr 29;52(11):6298–316. doi: 10.1093/nar/gkae313 (PMC11194086; doi:10.1093/nar/gkae313)
Supplement: gkae313_Supplemental_Files [file gkae313_supplemental_files.zip › Supplementary Materials.pdf]

Supplementary Materials

Senescence of human pancreatic beta cells enhances functional maturation through chromatin reorganization and promotes interferon responsiveness

Milan Patra, Agnes Klochendler, Reba Condiotti, Binyamin Kaffe, Sharona Elgavish, Zeina Drawshy, Dana Avrahami, Masashi Narita, Matan Hofree, Yotam Drier, Eran Meshorer, Yuval Dor and Ittai Ben-Porath

Supplementary materials include 6 supplementary Figures and 6 supplementary Tables

Figure S1

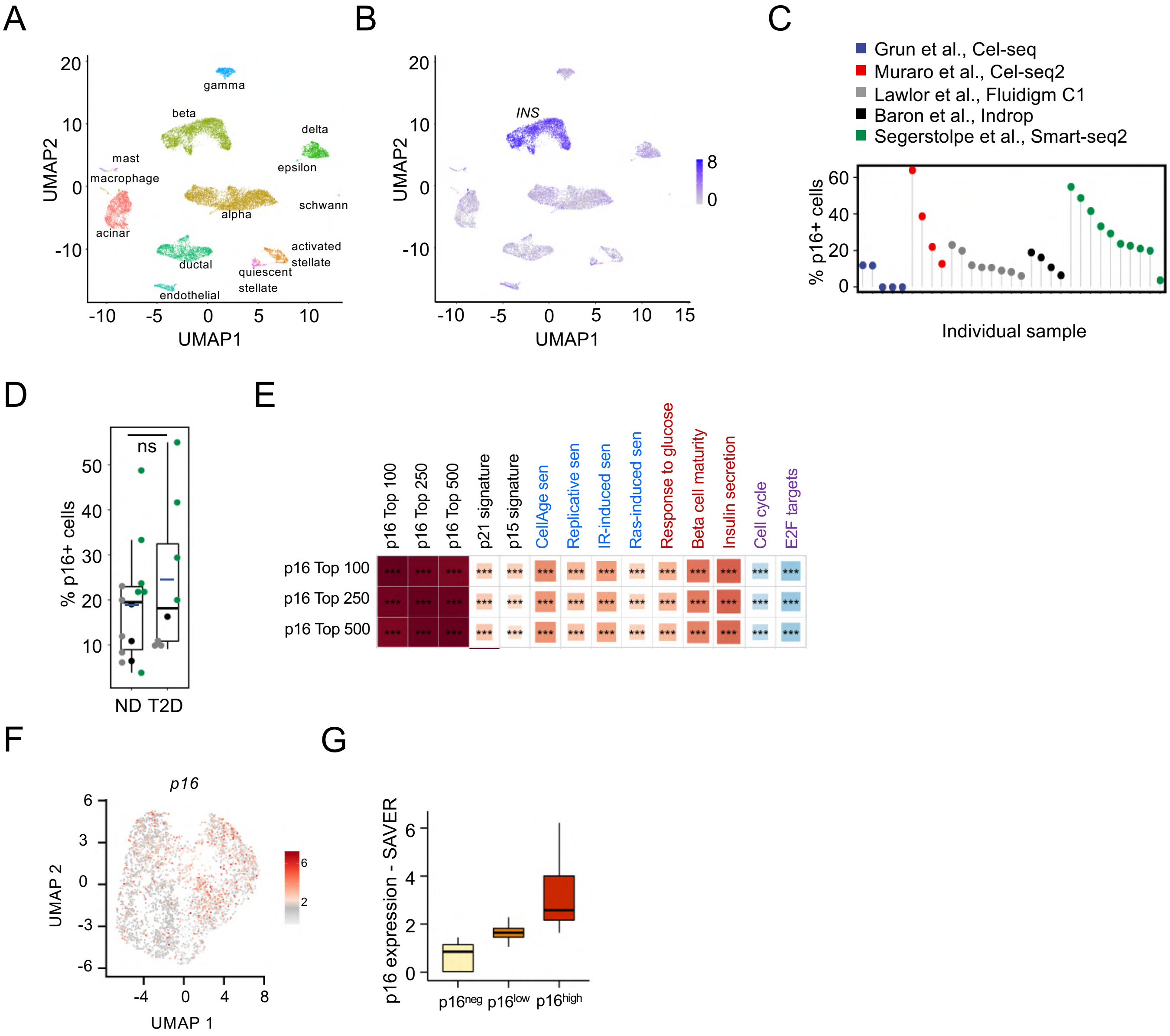
A
B
C
D
E
F
G
gamma
beta
delta
mast
macrophage
epsilon
alpha
schwann
acinar
activated stellate
ductal
quiescent stellate
endothelial
UMAP1
UMAP2
INS
Grun et al., Cel-seq
Muraro et al., Cel-seq2
Lawlor et al., Fluidigm C1
Baron et al., Indrop
Segerstolpe et al., Smart-seq2
% p16+ cells
Individual sample
ns
ND T2D
p16 Top 100
p16 Top 250
p16 Top 500
p21 signature
p15 signature
CellAge sen
Replicative sen
IR-induced sen
Ras-induced sen
Response to glucose
Beta cell maturity
Insulin secretion
Cell cycle
E2F targets
p16
UMAP 1
UMAP 2
p16 expression - SAVER
p16neg p16low p16high

Figure S1. Analysis of single-cell transcriptomes of human pancreatic beta cells. A) UMAP dimensionality plot of 14892 pancreatic cells from published datasets. Cluster labels indicate cells type. **B)** Same plot as in a, showing insulin expression levels (*INS*, blue). **C)** Percentage of p16-expressing cells in individual subjects (dots). Colors and legend indicate the original study in which each subject was profiled, and the transcriptome method used. **D)** Percentage of p16-expressing cells in non-diabetic (ND) and T2D patient samples. Boxes indicate interquartile values, black line indicates median and blue line indicates mean of all samples. Colors indicate original study, as in panel c. ns – non-significant, *t* test. **E)** Correlation matrix as in Figure 1K comparing the p16-associated signature comprised of the top 100 most correlated genes with p16 to signatures including the top 250 or top 500 most correlated genes. **F)** UMAP showing p16 levels in individual beta cells as in Figure 1D, following SAVER imputation correction. **G)** p16 levels in $p16^{neg}$ (<30th percentile), $p16^{low}$ (30-70th percentile) and $p16^{high}$ (>70th percentile) cell subgroups as defined using SAVER and used in Figure 1L,N, 2C,D.

Figure S2

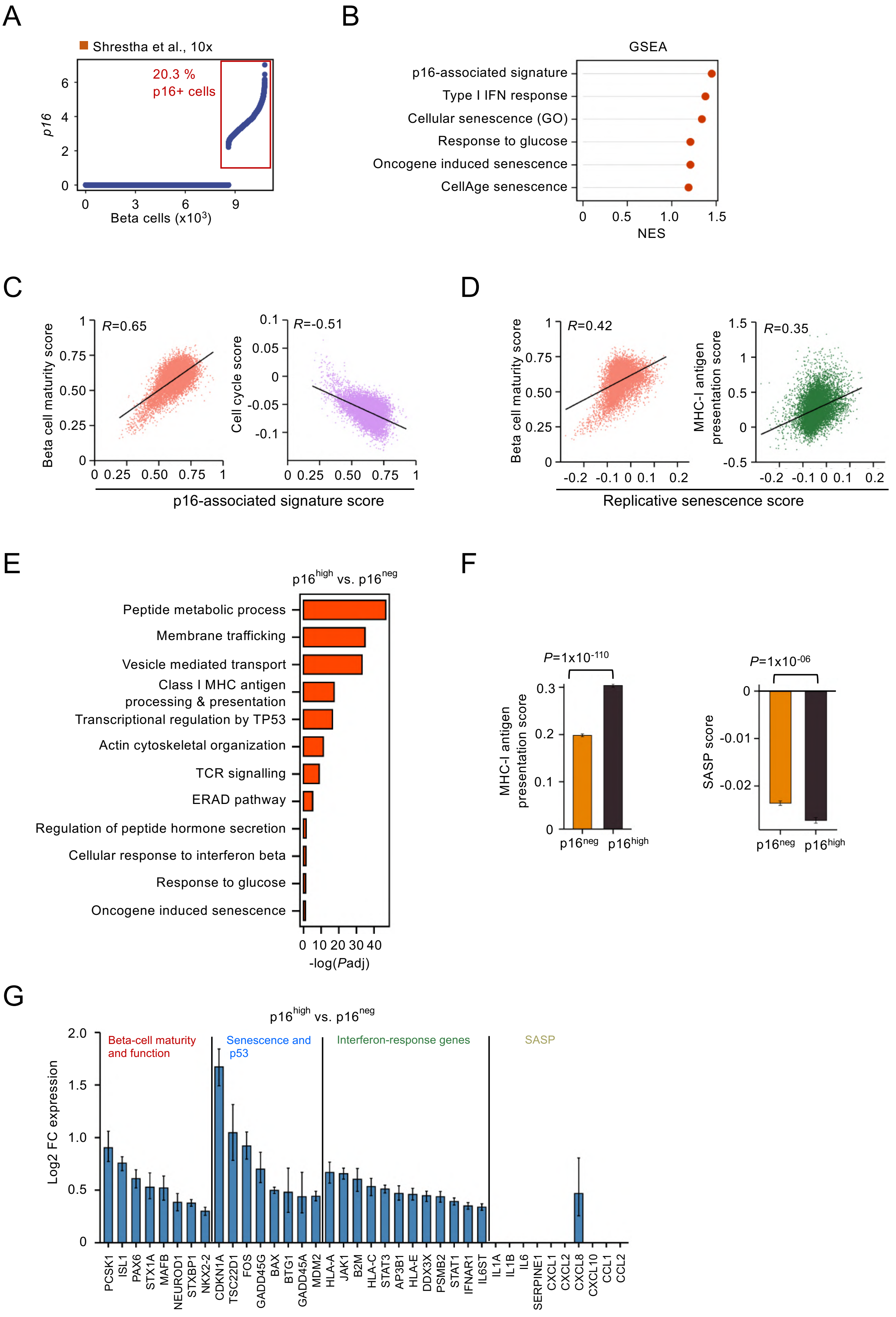

A
Shrestha et al., 10x
20.3 %
p16+ cells
p16
Beta cells (x10³)
B
GSEA
p16-associated signature
Type I IFN response
Cellular senescence (GO)
Response to glucose
Oncogene induced senescence
CellAge senescence
NES
C
R=0.65
R=-0.51
Beta cell maturity score
Cell cycle score
p16-associated signature score
D
R=0.42
R=0.35
Beta cell maturity score
MHC-I antigen presentation score
Replicative senescence score
E
p16high vs. p16neg
Peptide metabolic process
Membrane trafficking
Vesicle mediated transport
Class I MHC antigen processing & presentation
Transcriptional regulation by TP53
Actin cytoskeletal organization
TCR signalling
ERAD pathway
Regulation of peptide hormone secretion
Cellular response to interferon beta
Response to glucose
Oncogene induced senescence
-log(Padj)
F
P=1x10-110
P=1x10-06
MHC-I antigen presentation score
SASP score
p16neg
p16high
G
p16high vs. p16neg
Beta-cell maturity and function
Senescence and p53
Interferon-response genes
SASP
Log2 FC expression
PCSK1
ISL1
PAX6
STX1A
MAFB
NEUROD1
STXBP1
NKX2-2
CDKN1A
TSC22D1
FOS
GADD45G
BAX
BTG1
GADD45A
MDM2
HLA-A
JAK1
B2M
HLA-C
STAT3
AP3B1
HLA-E
DDX3X
PSMB2
STAT1
IFNAR1
IL6ST
IL1A
IL1B
IL6
SERPINE1
CXCL1
CXCL2
CXCL8
CXCL10
CCL1
CCL2

Figure S2. p16^{high} human beta cells display elevated levels of senescence, functional maturation and interferon-response genes in a validation transcriptome dataset. A) p16 (*CDKN2A*) mRNA expression levels in 10,787 single pancreatic beta cells from Shrestha et al. (27), obtained from 5 non-diabetic donors and profiled by 10x Genomics. **B)** Gene-set enrichment analysis (GSEA) of genes ranked by their correlation to p16 expression (SAVER) across beta cells in the same data set. x axis indicates normalized enrichment scores (NES) of gene sets in positively correlated genes (Padj<0.2). **C)** Plots indicating the expression score of the p16-associated signature (x axis) in individual beta cells (dots) relative to scores of other indicated gene signatures in the same cells. R values indicate Pearson correlation between the signatures across cells. $P<10^{-15}$ for all correlations. **D)** Same analysis as in C, with the replicative senescence signature in the x axis. $P<10^{-15}$. **E)** Gene sets upregulated in p16^{high} (n=3235) relative to p16^{neg} (n=3201) beta cells. x axis indicates -log(Padj) (Metascape). **F)** Mean score of IFN-response and SASP gene expression signatures in p16^{high} versus p16^{neg} beta cells. Values indicate mean score of indicated gene signature ±SEM, *t* test. **G)** mRNA levels of indicated genes in p16^{high} versus p16^{neg} beta cells. Values indicate mean of log2 fold-change (FC) of senescent versus non-senescent. Error bars represent 95% confidence interval.

Figure S3

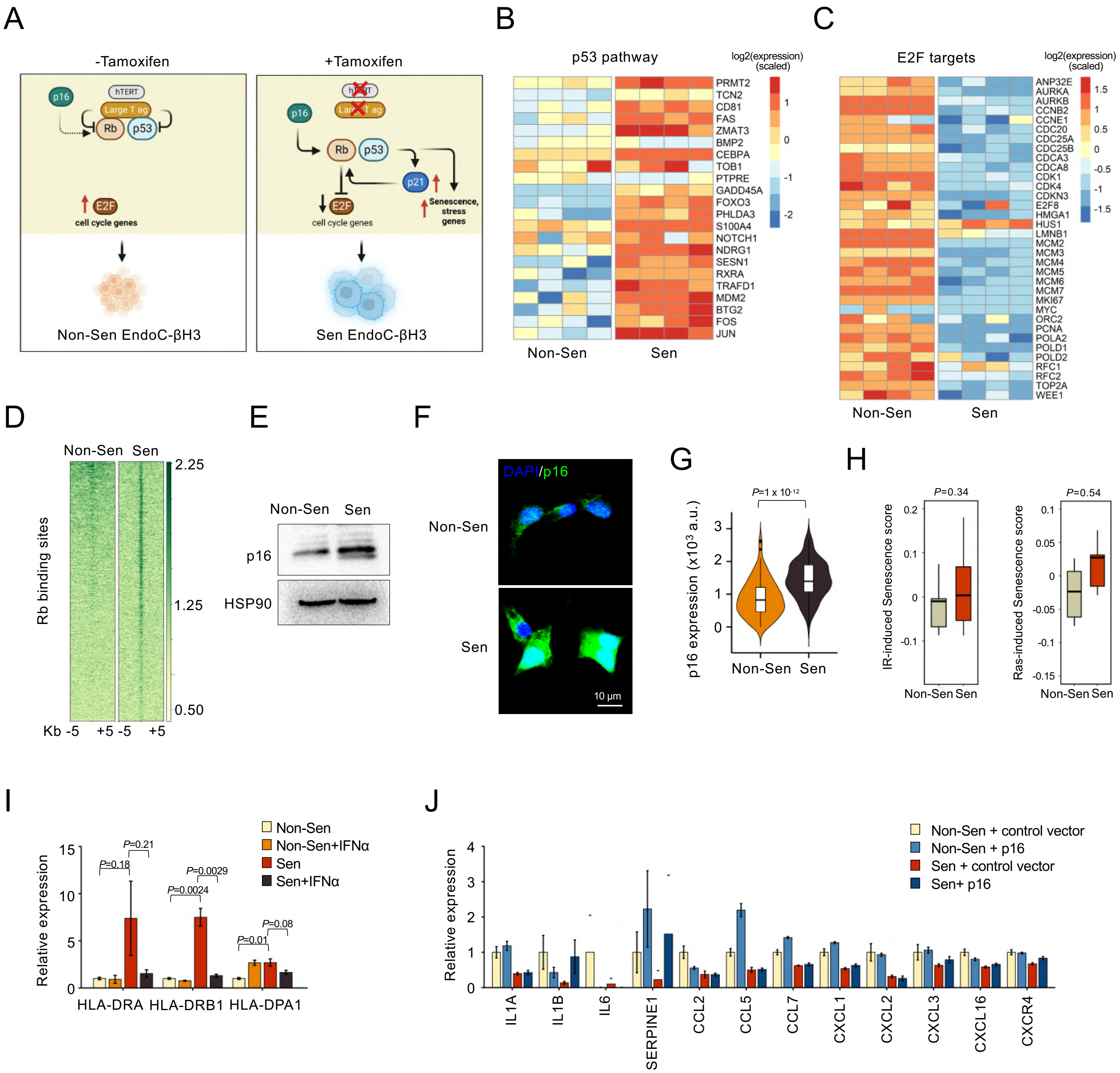

A
-Tamoxifen
+Tamoxifen
p16
hTERT
Large T ag
Rb
p53
p21
E2F
cell cycle genes
Senescence, stress genes
Non-Sen EndoC-βH3
Sen EndoC-βH3
B
p53 pathway
log2(expression) (scaled)
PRMT2
TCN2
CD81
FAS
ZMAT3
BMP2
CEBPA
TOB1
PTPRE
GADD45A
FOXO3
PHLDA3
S100A4
NOTCH1
NDRG1
SESN1
RXRA
TRAFD1
MDM2
BTG2
FOS
JUN
Non-Sen
Sen
C
E2F targets
log2(expression) (scaled)
ANP32E
AURKA
AURKB
CCNB2
CCNE1
CDC20
CDC25A
CDC25B
CDCA3
CDCA8
CDK1
CDK4
CDKN3
E2F8
HMGA1
HUS1
LMNB1
MCM2
MCM3
MCM4
MCM5
MCM6
MCM7
MKI67
MYC
ORC2
PCNA
POLA2
POLD1
POLD2
RFC1
RFC2
TOP2A
WEE1
Non-Sen
Sen
D
Non-Sen Sen
Rb binding sites
2.25
1.25
0.50
Kb -5 +5 -5 +5
E
Non-Sen Sen
p16
HSP90
F
DAPI/p16
Non-Sen
Sen
10 μm
G
P=1 x 10-12
p16 expression (x10³ a.u.)
Non-Sen Sen
H
P=0.34
IR-induced Senescence score
P=0.54
Ras-induced Senescence score
Non-Sen Sen
I
Relative expression
Non-Sen
Non-Sen+IFNα
Sen
Sen+IFNα
P=0.18
P=0.21
P=0.0024
P=0.0029
P=0.01
P=0.08
HLA-DRA HLA-DRB1 HLA-DPA1
J
Relative expression
Non-Sen + control vector
Non-Sen + p16
Sen + control vector
Sen+ p16
IL1A
IL1B
IL6
SERPINE1
CCL2
CCL5
CCL7
CXCL1
CXCL2
CXCL3
CXCL16
CXCR4

Figure S3. Senescence induction in EndoC-βH3 cells – molecular pathway activation. A) Diagram (BioRender) illustrating senescence induction in EndoC-βH3. In the absence of tamoxifen, hTERT and SV40 Large T-antigen are expressed, the latter binding and inhibiting Rb and p53. p16 is expressed but cannot activate Rb. Upon tamoxifen treatment, CreER excises hTERT and Large T-antigen, releasing p53 and Rb from inhibition. Rb binds chromatin to silence E2F targets and the cell cycle, and p53 activates its transcriptional targets, including p21, which further activates Rb. Together this induces senescence. **B,C)** Heat maps representing scaled log2 expression levels of p53 pathway (B) and E2F target genes (C) in non-senescent and senescent cells, measured by mRNA-seq. Columns indicate replicate samples. **D)** Rb binding in non-senescent and senescent EndoC-βH3 cells as measured by ChIP-Seq. Shown are loci with increased binding. **E)** Western blot of p16 protein in control and senescent EndoC-βH3 cells. **F)** Control and senescent cells stained for p16. **G)** Distributions of p16 protein expression in non-senescent (n=126) and senescent (n=112) cells, based on cell staining as in F. Fluorescence signal intensity of p16 is represented in arbitrary units (a.u.), *t* test. **H)** Scores of indicated senescence signatures in non-senescent and senescent EndoC-βH3 cells. Boxes indicate interquartile values with indicated median of n=6 replicates, *t* test. **I)** mRNA levels of indicated HLA-II encoding genes in non-senescent and senescent EndoC-βH3 cells, untreated or treated with IFNα, measured by qRT-PCR. Mean of n=3 experimental repeats ±SEM, normalized to untreated non-senescent cells, *t* test. **J)** mRNA levels of indicated SASP genes in non-senescent and senescent EndoC-βH3 cells expressing a control vector or a p16 overexpression vector, measured by qRT-PCR. Mean of n=3 experimental repeats ±SEM, normalized to control non-senescent cells. No genes show statistically significant increases in the senescent cells.

Figure S4

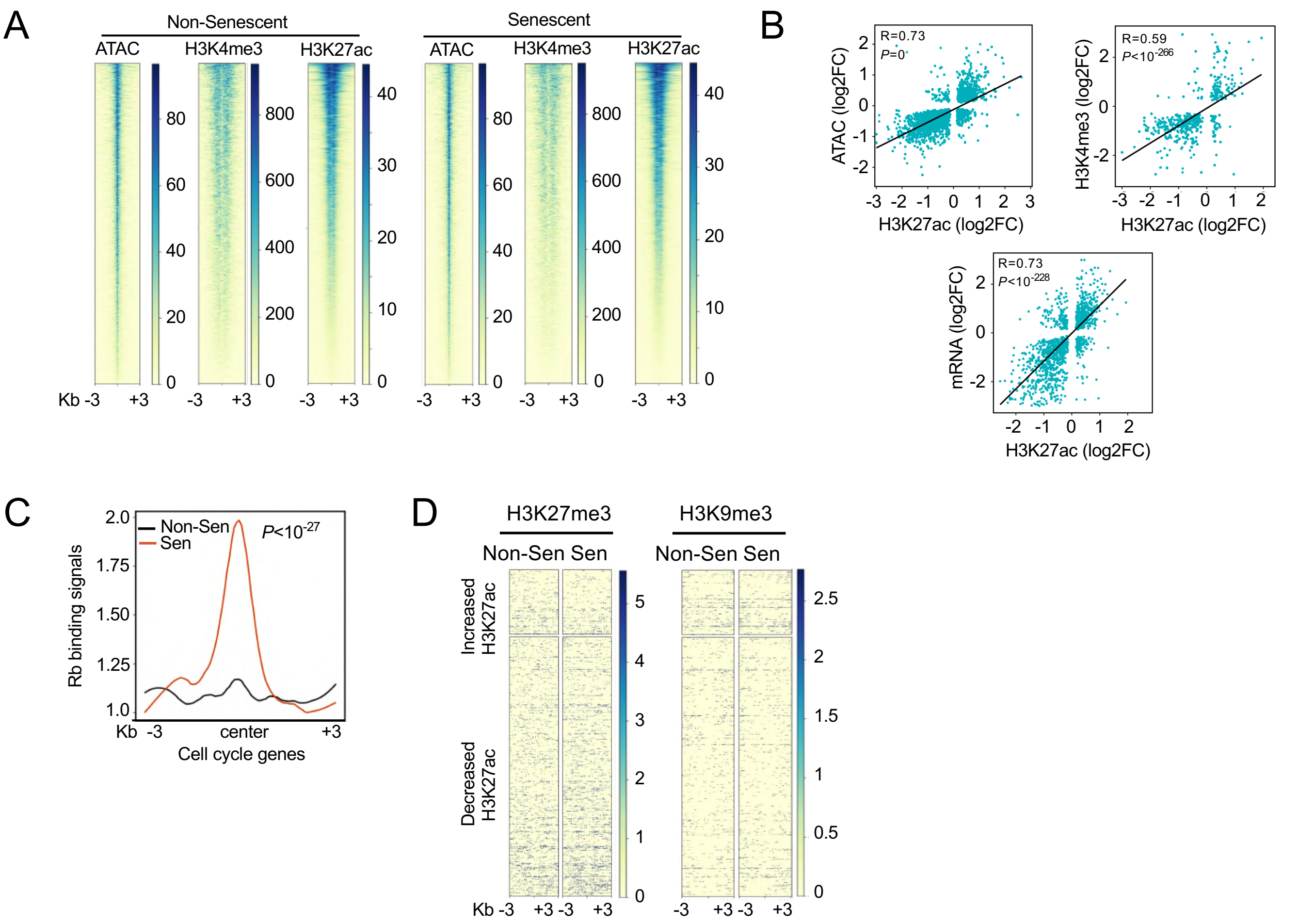
A
Non-Senescent
Senescent
ATAC
H3K4me3
H3K27ac
Kb -3
+3
B
R=0.73
P=0
ATAC (log2FC)
H3K27ac (log2FC)
R=0.59
P<10-266
H3K4me3 (log2FC)
R=0.73
P<10-228
mRNA (log2FC)
C
Non-Sen
Sen
P<10-27
Rb binding signals
center
Cell cycle genes
D
H3K27me3
H3K9me3
Non-Sen Sen
Increased H3K27ac
Decreased H3K27ac

Figure S4. Promoter chromatin marks in senescent EndoC-βH3 cells. A) Heat maps showing binding of activation chromatin marks – ATAC-Seq (accessibility), H3K4me3 and H3K27ac – on active promoters (n=9289) in senescent and non-senescent EndoC-βH3 cells. Shown are 6Kb regions spanning TSS peaks. All TSS ±2kb regions were filtered sequentially for the presence of all three activation marks. **B)** Correlations between H3K27ac binding level change in individual promoters upon senescence (x axes) and their change in ATAC-seq signal, H3K4me3 binding, or mRNA expression of the associated gene (y axes). Values indicate -log2 fold-change (log2FC) in senescent versus non-senescent cells. R indicates Spearman's correlation between indicated changes, with corresponding correlation *P* value. **C)** Aggregate plot of Rb binding near cell-cycle genes in non-senescent (black) and senescent (red) cells. x axis indicates position from -3Kb to +3Kb around binding peaks. y axis indicates normalized reads. *t* test on binned values. **D)** Heat maps showing binding of repressive chromatin marks – H3K27me3 and H3K9me3 – on promoters which show differential binding of the activation mark H3K27ac in senescent versus non-senescent EndoC-βH3 cells.

Figure S5

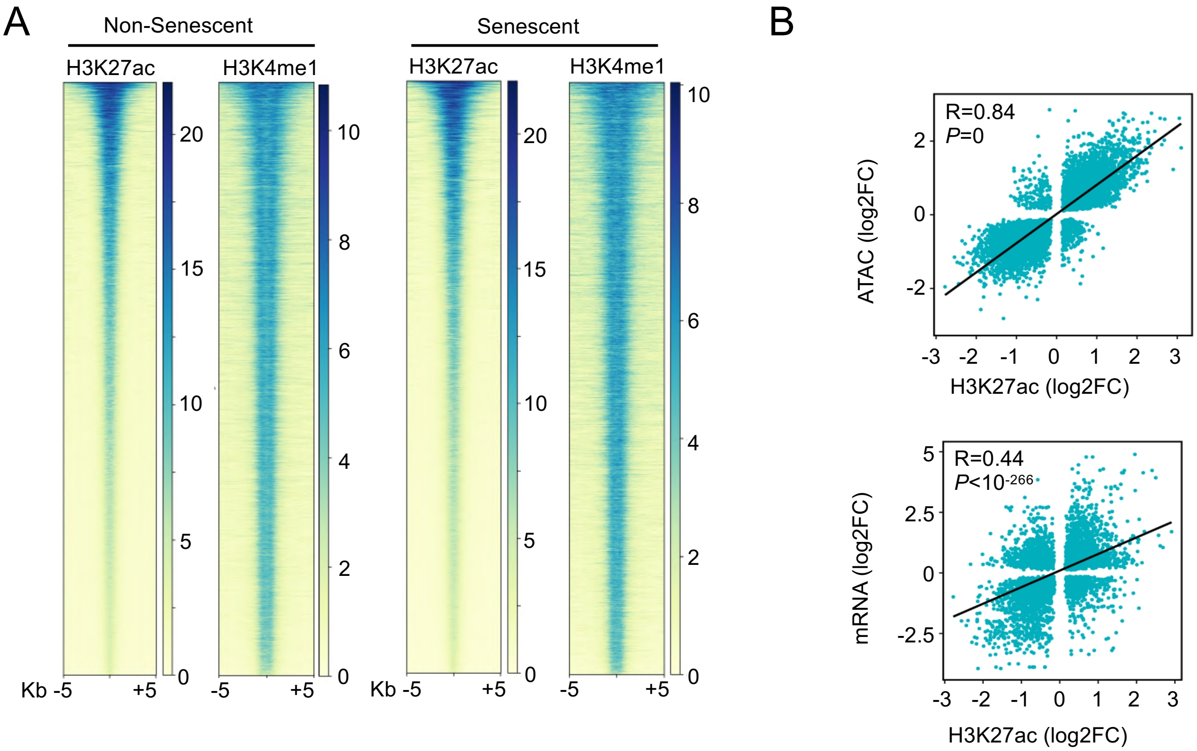
A
Non-Senescent
Senescent
H3K27ac
H3K4me1
H3K27ac
H3K4me1
Kb -5
+5
B
R=0.84
P=0
ATAC (log2FC)
H3K27ac (log2FC)
R=0.44
P<10-266
mRNA (log2FC)
H3K27ac (log2FC)

Figure S5. Gene enhancers in non-senescent and senescent EndoC-βH3 cells. A) Heat maps of loci bound by both H3K27ac and H3K4me1 in non-senescent and senescent EndoC-βH3 cells, identifying gene enhancers. **B)** Correlations between H3K27ac binding level change in individual enhancers upon senescence (x axes) and their change in ATAC-Seq signal (top) or in mRNA levels of associated gene (bottom). Shown are enhancers with changed H3K27ac binding level. Axes indicate log2 fold-change (log2FC) in senescent versus non-senescent cells. R indicates Spearman's correlation of change, with corresponding correlation *P* value.

Figure S6

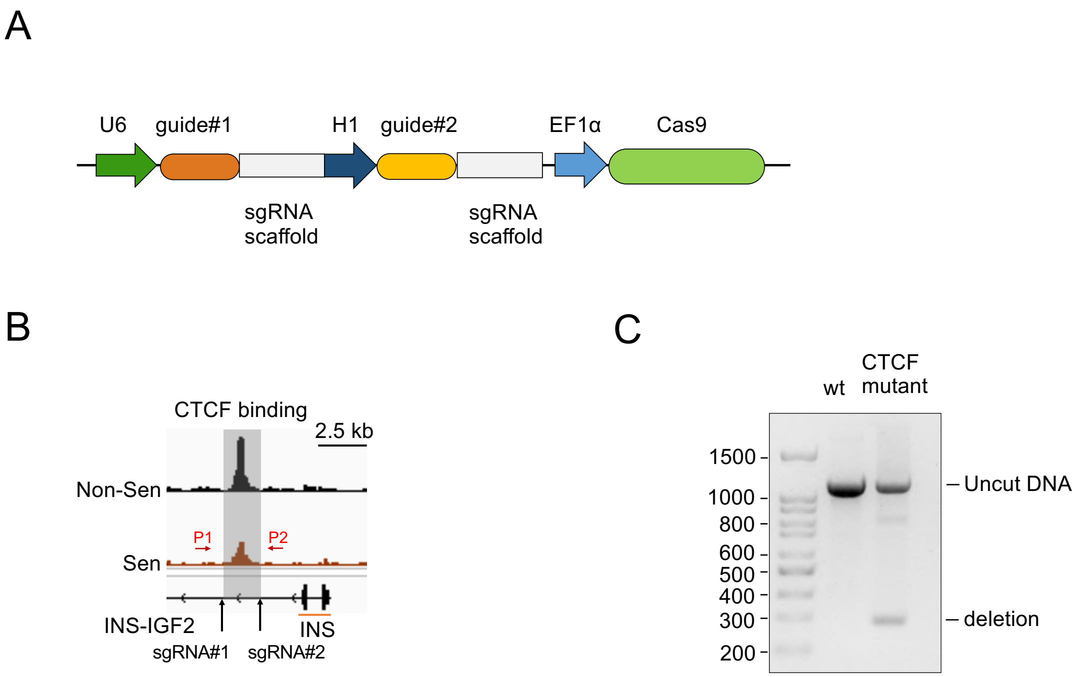
A
U6
guide#1
H1
guide#2
EF1α
Cas9
sgRNA scaffold
sgRNA scaffold
B
CTCF binding
2.5 kb
Non-Sen
P1
P2
Sen
INS-IGF2
INS
sgRNA#1
sgRNA#2
C
wt
CTCF mutant
1500
1000
800
600
500
400
300
200
Uncut DNA
deletion

Figure S6. CRISPR/Cas9-mediated deletion of CTCF binding site in the *INS* gene. A) Schematic representation of the lentiviral construct carrying Cas9 and the two guides targeting genomic sites spanning the 823 bp locus containing the CTCF binding site near the *INS* gene. **B)** Location of the CTCF-bound region (grey highlight) near the *INS* gene that was targeted for deletion. CTCF binding levels in non-senescent and senescent EndoC-βH3 cells are shown. Black arrows indicate targets of sgRNAs. P1 and P2 indicate approximate locations of PCR primers used to test deletion efficiency. **C)** PCR of genomic DNA from control (sgRFP-expressing) EndoC-βH3 cells and cells targeted for CTCF site deletion, using primers spanning the deletion site (P1, P2). The wt allele produces the observed 1116 bp product, whereas the deleted allele produces a 293 bp product. Quantification indicates 40-50% deletion efficiency in the targeted cells.

Table S1. Human samples used for section stains and FACS analysis. Related to Figures 1,2.

Table S2. Human samples used in original scRNA-seq studies. Related to Figure 1.

Table S3. Genes sorted by their correlation with p16 expression across beta cells. Related to Figure 1.

Table S4. Gene sets used in the study for enrichment analyses. Related to Figures 1-5.

Table S5. Gene signatures used for calculation of signature scores. Related to Figures 1-3.

Table S6. List of oligonucleotides. Related to Methods.
